# Surfaceome Capture by Multiplex Biotinylation Enables Enhanced Identification of Cell Surface Proteins by Mass Spectrometry

**DOI:** 10.1016/j.mcpro.2026.101572

**Published:** 2026-04-22

**Authors:** Ana Levi, Tommy Shields, Irbaz I.Badshah, Vinothini Rajeeve, Pedro R. Cutillas

**Affiliations:** 1 Cell Signalling & Proteomics Group, Barts Cancer Institute, Queen Mary University of London, John Vane Science Centre, London, United Kingdom; 2 Instituto de Parasitología y Biomedicina López-Neyra, Consejo Superior de Investigaciones Científicas, CSIC, Granada, Spain

**Keywords:** cancer, drug target, leukaemia, immunotherapy, surfaceomics, quantitative proteomics

## Abstract

A key goal of surfaceomic discovery technologies is to comprehensively interrogate the cell surface proteome to identify targets for immunotherapy development. Considerable progress has made in the application of surfaceomics to profile cancer cells. However, challenges still exist in the sensitivity of current surfaceomic approaches, which consequently, are restricted to the analysis of cell lines or primary tumour material that contain a relatively large number of cells. In addition, since current approaches are based on labelling a single functional group on polypeptides, it is not clear if these recover the full spectrum of proteins present on cell surfaces. To circumvent these limitations, we developed a biotinylation-based combinatory approach for isolating a more diverse group of proteins facing the cell periphery. Our proposed approach, named Surfaceome Capture by Multiplex (SUCAM) biotinylation consists of “multiplexing” biotin reagents to enable for multiple-functional group derivatisation of the surface proteome. LC-MS/MS is then used to identify and characterize proteins pulled down by streptavidin magnetic beads. We found that SUCAM identified more plasma membrane and cell surface proteins than methods based on labelling with single reagents, leading to enhanced identification of cell surface proteins from just 1.2 million cells. Replicate experiments revealed that surfaceomic proteins could be quantified with good precision across repeats (coefficient of variation of 1.7% on average). Application of the approach to a panel of leukaemia cell lines identified well-known leukemic cell surface antigens, as well as proteins with hitherto uncharacterised roles in this disease. SUCAM is a complementary biotinylating strategy that will facilitate surfaceomic profiling for discovery of therapeutic targets in haematological and solid tumours.

## Highlights

• Surfaceome Capture by Multiplex biotinylation (SUCAM) is optimized to detect therapeutic targets.
• SUCAM is based on “multiplexed” biotinylation against diverse functional groups.
• SUCAM enables comparatively efficient and selective surfaceome enrichment.
• SUCAM shows robust and quantitative precision using just 1.2 million cells as input.
• Application in leukemic cells identified potential new targets for immunotherapy.

## Graphical Abstract

**Figure:**
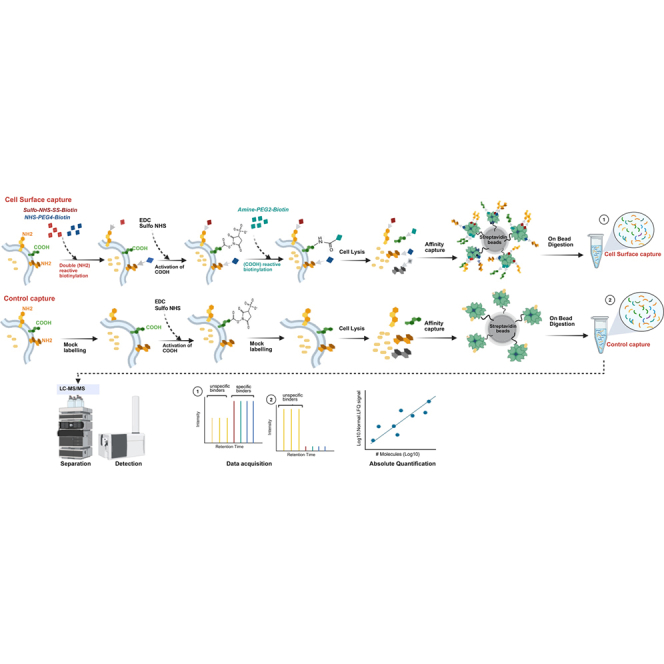

## In Brief

We aimed to advance existing cell-surface protein enrichment methods by multiplexing the biotinylation of protein functional groups. The resulting approach, named Surfaceome Capture by Multiplex biotinylation, enriched a larger and more diverse set of surface proteins from low-input material than approaches based on single biotinylation. Application to leukemic cells identified canonical surface antigens as well as proteins with previously uncharacterized roles in this malignancy. Our findings highlight the utility of SUCAM for the discovery of immunotherapeutic targets.

Cell surface and plasma membrane proteins (PMPs) regulate the organismal and cell physiology by mediating intercellular communication and intracellular signal transduction, allowing cells and tissues to adapt to changes in their extracellular environment, and thus controlling essentially all cell biological functions (1). Despite the PMPs constituting a vast scope of the human genome, there remains a large fraction to be fully characterised. Exploring the expression patterns and functionality of these proteins in healthy and disease situations is important to discover their fundamental functions in normal cellular physiology and disease mechanism (2). Cell surface proteins are also the target for new immunotherapies such as antibody drug conjugates (ADCs) and chimeric antigen receptor (CAR) T cells and are thus potentially valuable for targeted drug interventions (3, 4). Approximately 22% of all proteins coded by the human genome are found at the cell surface, but such fraction of the proteome is underrepresented in the literature and in high throughput proteomic analyses (5). Addressing this knowledge gap is expected to assist in the elucidation of aberrant alterations in surface protein expression, modification and interactions directly related to human diseases (6). Thereby, a more comprehensive and thorough understanding of the surfaceome will assist in identifying surface proteins for the development of novel targeted immunotherapies.

Mass spectrometry (MS)–based proteomics has become an increasingly valuable approach for studying expression patterns of PMPs and cell surface proteins (7, 8, 9), making this a very powerful technology to globally and systematically characterise cell surface proteins for identifying cancer biomarkers and drug targets. Therefore, although comprehensive characterization of the surfaceome remains extraordinarily challenging, proteomic approaches are starting to reveal the surfaceome of different tumour types (10, 11, 12). To specifically and comprehensively characterize proteins only located on the cell surface, the extraction, enrichment and purification of integral proteins are prerequisites for LC-MS/MS based identification and quantification, and this still represent a challenge in the process of defining the PM proteome (13, 14). This is because of the complexity of the biochemical, topographical and structural properties of the PM proteins, including (1) the hydrophobicity of the domains that transverse the membrane (2) the generally low abundant nature of these proteins compared with the other cellular proteins and (3) and problems associated with the separation of PM proteins from those of other organelles (15).

The difficulty is heightened by the heterogeneous character of these proteins and their specific physical-chemical properties (1). In the case of transmembrane proteins, PMPs vary in the number of membrane-spanning helices (from one to 15), the ratio of membrane hydrophobic embedded domains to soluble intra- and extracellular portions and the number of modifications, such as glycosylation’s, that contribute to protein solubility (16). This represents an obstacle in the identification of integral membrane proteins and cell surface proteins with highly hydrophobic character. A further obstacle is the limitation in the number of cells required, particularly for analysis of primary samples.

To help overcome these obstacles in characterising the complexity of cell surface proteins, a number of MS-based methods for PMP and surfaceome enrichment have been developed (17, 18). A family of such methods are based on the chemical modifications of cell surface proteins at sites of glycosylation for cell surface capture (CSC) (19). The CSC method, which is based on the chemical derivatization of glycated residues with a biotin linker, has been used to profile the surfaceome of a range of cell lines, primary cells and tissues, resulting in a mass spectrometry derived Cell Surface Protein Atlas (CSPA) with 1492 human cell surface glycoproteins (20). CSC identifies N-linked glycosylation sites (N x S/T/C motif) by detecting a specific 0.984 Da mass shift in the MS spectra, caused by the conversion of glycosylated asparagine to aspartic acid during enzymatic release. It overcomes the limitations of traditional, non-specific membrane protein isolation by focusing exclusively on proteins with extracellular carbohydrate moieties, which constitute the majority of the cell surface proteome. As an alternative to CSC, Weekes *et al* developed an aminooxy-biotinylation procedure for capturing sialylated PM glycoproteins, resulting in superior protein sequence coverage and improved PMP enrichment (18). Sialic acid resides on the termini of both N- and O-glycans, making sialylated capturing highly specific to terminal modifications, whereas N-glycoprotein capture focuses on the core structure attached to asparagine (Asn). Some disadvantages of both these studies include a requirement for a relatively large amount of starting material (nearly 10^8^cells or 200 mg – 1 g of tissue), as well as the potential loss of glycan structures by oxidation, leading to low sensitivity.

In a recent important development, R. Thorup *et al* compared the performance of two distinct cell surface proteomics strategies applicable on cryopreserved and tissue-derived samples using 1 x 10^6^ to 5 x 10^6^ HEK293 T cells (21). The N-Glycopeptide Enrichment (NGE) strategy involves the selective introduction of aldehydes into sialic acids or into terminal N-acetylgalactosamine by periodate oxidation, followed by 5-methoxyanthranilic acid (5-MA) as a catalyst for generating aldehyde-based, biorthogonal conjugation to alkoxyamine–PEG4–biotin. NGE was benched marked against the WGA-HRP strategy, which involves using the lectin WGA to bind specifically to N-acetylglucosamine (GlcNAc) and sialic acid residues on the cell surface, thus targeting the HRP enzyme to the outer membrane. Once bound to the membrane, HRP utilizes hydrogen peroxide and biotin-tyramide to catalyze the formation of a biotin-phenoxyl radical, which then labels nearby tyrosine residues on surface proteins. Both strategies enabled surfaceome profiling from cryopreserved and low input samples (<1 x 10^6^cells), isolating 200 CSPs at the lowest input (1 x 10^5^cells) and ∼500 CSPs at 2.5 x 10^6^cells from both fresh and cryopreserved single-cell suspensions obtained from fresh endometrial biopsies.

As an alternative to glycan-based CSC, other chemical biotinylation methods have been successfully used to capture cell surface proteins irrespective of their posttranslational modification status. In such approaches, biotin reagents are derivatised with reagents that label primary-amines (-NH2), thus allowing covalent attachment of proteomes and subsequently enrichment using avidin-bound resins or beads by affinity purification. Primary amines (-NH2) are favourable targets for biotin derivatisation because they are the most common moieties in proteins, with lysines accounting for approximately 6% of all amino acid residues, and being abundantly present on the surfaces of native protein tertiary structures, making them easily accessible for biotin labelling (17). Furthermore, primary amines are the most nucleophilic of all the protein functional groups, making them a favourable target for biotin derivatisation. Additionally, the amine group at the protein N-terminus can also be labelled. To date, a variety of NHS-ester derivatives have been developed for cell surface protein enrichment, many of which are commercially available. NHS-ester reagents can vary in length, solubility, cell permeability, and cleavability. Regarding cell permeability, sulfo-NHS-SS-biotin with a negatively charged sulfate group is reported to be membrane impermeable, a favourable characteristic that prevents the unwanted labelling of intracellular proteins. It is noteworthy, however, that the use of NHS-PEG4 carries a potential risk of contamination from intracellular proteins due to its hydrophobic long chain. Furthermore, intracellular proteins leaked from dead cells may also be biotinylated with NHS esters, thus confounding surfaceomic data with background proteins that are not PMPs or located on the cell surface.

A potential limitation of all NHS-ester reagents that label free amines is that surface proteins with few or no exposed amines may be overlooked in surfaceomic experiments based on such chemical labels. As an alternative to such reagents, carboxyl reactive conjugation agent label surface exposed carboxyl groups on aspartic acid (Asp), glutamic acid (Glu), and protein C-termini. One advantage of carboxyl labelling is the frequencies of Asp and Glu residues on the extra-cellular Transmembrane Proteins (TMPS), which some studies have found to be larger than that of Lys residues (22). However, despite the potential for carboxyl-based biotinylation for surfaceome enrichment, these reagents have not been fully explored for this purpose.

Despite these advances, however, current methods for surfaceome analysis have limitations in terms of selectivity and sensitivity. Indeed, most published approaches require the use of several million cells as input material, limiting their utility to the analysis of primary tumours for which large number of cells can be obtained. There is also a need to systematically characterise the performance of surfaceomic methodologies carried out with low number of cells as input and explore the potential to combine different biotinylation reagents to broaden the physiochemical properties of proteins that can be characterised with these techniques. To address these challenges, we developed a biotinylation approach based on ‘’multi-plexing’’ unique biotin agents, name Surfaceome Capture by Multiplex (SUCAM) Biotinylation. We reasoned a formulation of “multiplexed” biotinylating reagents, containing multiple functional group reactivities, would enable a more efficient and selective labelling of cell surface proteins, thus broadening the coverage of the cell surface proteome.

## Experimental Procedures

### Combinatory Biotinylation of the Cell Surface Proteome Using Amine Reactive (NH2 Labelling) and Carboxyl Reactive (COOH Labelling)

Cells are washed twice with PBS+ (PBS supplemented with 0.1 mM CaCl2, 1 mM MgCl2) pH 7.4 and once with PBS + pH 8.0 and then incubated with 2 mM EZ-Link Sulfo-NHS-SS-biotin (21,331; Pierce) and 2 mM EZ link NHS-PEG4 biotin (21,330; Pierce) in PBS + pH 8.0 for 30 min at 4 °C with gentle shaking. The control consists of a mock pull-down, by which cells are initially treated the same except that the 2 mM treatment with biotin one and biotin two was replaced with vehicle (PBS). Cells were washed x1 with TBS pH 7.5, and x2 with in PBS pH 8.0 (to quench and wash off residual biotin, respectively).

To proceed with carboxyl labelling, cells were washed with PBS + pH 5.7 and then preincubated with 2 mM EDC and 5 mM NHS-Sulfo in PBS pH 5.7 for 15 min at 4 °C with gentle shaking. After preincubation, cells were washed with PBS + pH 8.0 and subsequently incubated with 2 mM carboxyl reactive biotin (EZ-Link-Amine-PEG2 Biotin (21,346; ThermoFisher) in PBS pH 8.0 for 40 min at 4 °C with gentle shaking. As control, we performed a mock pull-down, cells are treated the same except the 2 mM treatment with Biotin is replaced with PBS. After incubation with Amine PEG2 biotin, residual biotin was quenched with 100 mM glycine in PBS pH 8.0 and then washed twice with PBS pH 7.4.

### Aminooxy Biotin-Based Labelling of N-Glycoproteins

Hepatocellular carcinoma (HCC) derived cell line, JHH4 was cultured at 2.2 x 10^6^ per 10 cm cell culture and grown at 70 to 80% confluency. The cells monolayer was washed x2 with PBS (pH 7.4) and 5 ml 0.25% type one collagenase in PBS (pH 7.4) was added to the cell cultures and incubated at 37 °C for 5 to 20 min until the ECM was digested. The liberated cells were collected from the Petri dish to a centrifuge tube, then washed x2 with 5 ml ice-cold PBS (pH 7.4) to collect the remaining cells. These fractions were pooled into the same centrifuge tube and centrifuged at 300×*g* for 5 min at 4 °C to pellet the cells. Cell pellets were washed x2 with ice cold GlycoLink Coupling Buffer and centrifuged at 300×*g* for 5 min at 4 °C to pellet the cells. Cells were resuspended in GlycoLink Coupling buffer and 10 mM sodium metaperiodate was added to the cell mixture and incubated at 4 °C for 10 min in the dark. Periodate solution was aspirated and washed cells x3 with GlycoLink Coupling buffer, after which an additional 2 ml of GlycoLink Coupling Buffer and 18 μl of aniline were added to the cells. Alkoxyamine-PEG4-biotin was prepared to a final concentration of 1 mM in GlycoLink Coupling Buffer and one part of prepared alkoxyamine-PEG4-biotin solution was added to nine parts oxidised sample (results in 0.1 mM alkoxyamine-PEG(n) biotin) and mixed for 1 h at room temperature. After 1 h, the reaction was stopped by the addition of 400 μl glycerol at a final concentration of 1 mM and the cells were incubated for 10 min in the dark at 4 °C and subsequently washed x3 with ice-cold PBS.

### Isolation of Biotinylated Proteins

Cells were lysed in modified RIPA buffer (50 mM Tris-HCl, 150 mM NaCl, 1 mM EDTA, 10 mM iodoacetamide, 0.2% SDS (v/w), 0.5% sodium deoxycholate, 1% Triton X-100, pH 8.0) containing protease inhibitors. Cell lysates were centrifuged at 15,000 x g for 5 min at 4 °C, the nuclear pellet was discarded, and supernatants were incubated with preconditioned Streptavidin Magnetic Beads (8817; Pierce) for 3 h to overnight at 4 °C. Unbound samples were collected, and beads were washed as follows; x 3 RIPA lysis buffer, x3 in Urea buffer (8 M urea in 0.1 M Tris/HCl pH 8.5), x two in 2M NaCl, x 3 with 50 mM Ammonium bicarbonate (AMBIC), x 1 with 25 mM AMBIC. Finally, the biotinylated proteins were digested on-beads at 37 °C with 2 μg Trypsin/Lys-C Protease Mix (Pierce) (1:50 w/w) overnight in 25 mM ammonium bicarbonate. We harvested the eluted digested peptides (clear supernatant without the beads). The beads were then rinsed with 50 ul of 25 mM ammonium bicarbonate, and this second tryptic fraction was pooled with the first one.

For each N-Glycoprotein enrichment reaction, cells were lysed in 500 μl lysis buffer containing 20 mM Tris pH 7.4, 150 mM NaCl, 1% Triton X-100, 10 mM β-glycerol phosphate, 1 mM EDTA, 1 mM EGTA, 5 mM iodoacetamide, 10 mM NaF and 0.1 mg/ml PMSF protease inhibitors and incubated at 4 °C for 2 h. The lysates were sonicated using Bioruptor with high intensity and pulsed settings (Cycles: 10–20, On: 30s, off: 35s). Lysates were centrifuged at 13,000 rpm for 10 min at 5^°^C and the supernatant recovered. High-capacity streptavidin-agarose resin was prepared by packing 250 to 300 μl resin into columns and washed x3 with binding buffer (0.1 M sodium phosphate, 0.15 M sodium chloride, pH 7.2). The resin was incubated with lysate for 60 min at 25 °C whilst shaking and then washed x3 with WB1 (0.4% SDS, 20 mM Tris, pH 7.5, 400 mM NaCl), x8 with WB2 (20 Mm Tris, pH 7.5 and 400 mM NaCl), x8 with WB3 (50 Mm TEAB, 2M Urea). 400 μl of 50 mM AMBIC/1.33 M Urea (∼9 pH) was added to the matrix which was then transferred to protein LoBind tubes (Eppendorf). The matrix was incubated with 45 mM DTT for 45 min and alkylated with 100 mM iodoacetamide for 30 min in the dark at RT. A total of 5 μg modified sequencing grade Trypsin (Promega) was added to the matrix and the biotinylated glycoproteins digested on beads at 25 °C with shaking @ 1400 rpm. Tryptic peptides were collected by centrifugation at 1000 x g for 1 min in Snap Cap spin columns. The resin was washed x1 with 400 μl of 50 mM AMBIC pH 8.8 and tryptic fractions were pooled. For additional PNGase F digestion, the resin was washed x3 with WB4 (50 Mm HEPES, pH 7.5) followed by incubation with 50 units of PNGase F in WB4 for 3 h at 37 °C. Peptides were eluted by centrifugation.

### Sample Preparation for Western Blotting

Biotinylated protein lysates were incubated with preconditioned Streptavidin Agarose Resin (20,357; Pierce) for 3 h to overnight at 4 °C. Unbound samples were collected, and beads were washed to remove non-specifically bound proteins using standard procedure. Biotinylated proteins were eluted from the beads with "mild" elution consisting of 0.4% SDS, 1% IGEPAL-CA630 and 25 mM free biotin at 70 °C for 5 min to minimize bead monomer release. The supernatant, containing the eluted proteins was collected following centrifugation at 1500 rpm for 5 min. Protein in the cell extracts was quantified by bicinchoninic acid analysis. Finally, 80 μl of four x sample buffer (200 mM Tris HCl pH 6.8, 40% glycerol, 8% SDS, 10 mM DTT, 0.04% bromophenol blue) was added to 60 μg of protein extract and heated at 95 °C (for full denaturation of streptavidin) or 70 C (for streptavidin to remain in its tetramer form) for 5 min. Samples were analysed in four to 12% precast commercial gels (NuPAGE Novex 4–12% Bis-Tris Midi Gel 1.0 mm) with NuPAGE MOPS SDS running buffer 20x. Electrophoresis was run at room temperature using a constant voltage. After electrophoresis, gels were washed in transfer buffer (10% methanol, 0.1% NuPAGE Antioxidant diluted in NuPAGE transfer buffer 20x) for 10 min. Then, separated proteins were transferred to a nitrocellulose membrane (iBlot Gel Transfer Stacks Nitrocellulose) for 13 min, applying a constant voltage. Non-specific binding was blocked with 5%BSA in TBS-T (0.1% Tween-20) and the membrane washed x3 with TBST (TBS + 0.1% Tween-20). The membranes were incubated for 14 h at 4 °C with primary antibody (Cell Signalling Technology). Following this, after several washes, membranes were incubated with secondary antibody against rabbit immunoglobulin conjugated with peroxidase (Anti-rabbit IgG, HRP-linked Antibody #7074, Cell Signalling Technology. Finally, membranes were incubated for 1 min with 1x SuperSignal West Pico ECL solution, which afforded a chemiluminescence reaction. The chemiluminescent signal is detected and recorded by exposure of the membrane to a light-sensitive film (Kodak X-ray film). A pre-stained protein marker was used to assign molecular weight to the isolated proteins (Invitrogen). Antibody affinity was then quantified using Image J (1.54r).

### Sample Preparation for Proteomics

The samples were acidified to 1% TFA and peptide desalting was performed by reverse-phase liquid chromatography on an Agilent Bravo Automated Liquid Handling Platform, using the peptide clean-up v3.0 protocol. Briefly, reverse phase S cartridges 12 (Agilent, 5 μl bed volume) were primed with 250 μl 99.9% acetonitrile (ACN) with 0.1%TFA 13 and equilibrated with 250 μl of 0.1% TFA at a flow rate of 10 μl/min. The samples were loaded (770 μl) at 20 μl/min, followed by an internal cartridge wash with 250 μl of 0.1% 15 TFA at a flow rate of 10 μl/min. Peptides were then eluted with 105 μl of 1M glycolic acid with 50% ACN, 5% TFA. After desalting, 150 μl of the elute is transferred to Lo-bind tubes and subjected to SpeedVac used to concentrate the samples overnight and then stored at −80 °C.

### Mass Spectrometry

Peptides were re-suspended in 20 μl of reconstitution (97% H2O, 3% ACN, 0.1% TFA, 8 50 fmol/μl-1 enolase peptide digest) and sonicated for 5 min at RT. Following a brief centrifugation, 4 μl was loaded onto a LC-MS/MS system. The LC-MS/MS platform consisted of a Dionex UltiMate 3000 RSLC coupled to Q Exactive Plus Orbitrap Mass Spectrometer (Thermo Fisher Scientific) through an EASY-Spray source operated as described before (23, 24).

### Peptide Identification from Tandem Mass Spectrometry Data

For peptide identification, Peak list files (MGFs) from RAW data were generated with Mascot Distiller v2.5.1.0 and loaded into Mascot Daemon (v2.8.0.1) search engine for matching of the MS/MS data to peptides. Supplemental Table S1 includes RAW file names linked to mzID files. Mascot Daemon (v2.8.0.1, in-house server) matched the MS/MS data to the Swissprot Database for *Homo sapiens* (SwissProt_Sep2014_2015_12.fasta) with the number of entries for the searches being 20,194 sequences, 181,677,051 residues). The FDR was set to ∼1% (peptide matches above homology or identity threshold). Searches had the following parameters: two trypsin missed cleavages, mass tolerance of ±10 ppm for the MS scans and ±25 mmu for the MS/MS scans, carbamidomethyl Cys as a fixed modification and oxidation of Met, PyroGlu on N-terminal Gln and deamidated (NQ) as variable modifications. Mascot calculated false discovery rate by comparing results against a decoy database.

### Peptide Quantification from MS1 Data

In-house developed Pescal (Peak Statistic Calculator) software was used to calculate m/z and retention time (tR) values for each identified peptide ion to generate extracted ion chromatograms (XICs). The software constructed XICs for all the peptides identified in at least one of the LC-MS/MS runs across all samples. XIC mass and retention time windows were ±7 ppm and ±1.5 min, respectively. Algorithms in Pescal then calculate area under the peak of the XICs in the generated arrays and return these values to Excel file for further analysis. The relative quantity of a peptide was calculated relative to the mean of peptide normalized ion intensities of this peptide across the samples to be compared. For the reported differentially expressed proteins at least one unique peptide assigned to them was also required. We accepted the returned protein identifications when Mascot scores were above the statistically significant threshold (expectancy <0.05 for individual peptides) and at least two peptides matched the identified protein.

### Bioinformatic Analysis

A bioinformatics pipeline that incorporates R algorithms for pathway analysis and interactive visualisations (https://github.com/CutillasLab/protools2/) was used for the quantitative processing of the proteomic data. Peak area normalisations were performed and then log2 scaled and cantered. Statistical differences between comparisons were calculated using the LIMMA function and then *p*-values adjusted for FDR using the Benjamini-Hochberg method. In some instances, Protools2 was used to conduct GO enrichment analysis of the proteomic data against the cellular component (CC) subset of ontologies obtained from Uniprot or the in silico surfaceome obtained from Bausch-Fluck *et al* (25). Proteins were considered significant if present in labelled samples with Log2FC > 0.5 and adjusted *p*-value <0.1 compared to the mock labelled control.

In other instances, ClusterProfiler (R) was used to conduct Gene set enrichment analysis (GSEA) and Over-representation Analysis (ORA) using Gene Ontology (GO) gene sets for biological process (BP), molecular function (MF), and CC on differentially expressed proteins (DEPs) in Biotin labelled versus Control comparison. We selected specific, pre-determined Log2FC and adjusted *p*-value thresholds for each experiment based on the specific experimental design, particularly considering the number of biological and technical replicates used. Gene set sizes were set to a minimum of three and a maximum of 350 with *p*-value adjustment using the Benjamini-Hochberg (BH) method (*p*< 0.05). Enriched pathways were visualized using DOSE (R) and ggplot2 (R) packages.

Pearson correlation was used to analyse the linear relationship between the five biotin conjugation methods from numeric variables (Log2FC.Biotin *versus* Control). Correlation analysis was conducted in R using the cor() function to calculate the Pearson correlation matrix. The results were visualized using the corrplot package, with the corrplot() function, to present the strength and direction of the correlations, allowing for easy identification of significant relationships. A positive correlation indicates that as one variable increases, the other also increases, while a negative correlation indicates that as one variable increases, the other decreases. Significance levels (*p*-values) were calculated using cor.test() ()` and were used to determine if the correlations were statistically significant.

### Experimental Design and Statistical Rationale

To support the development of SUCAM, we employed a label-free single-shot LC-MS/MS based analytical platform which overcomes the issue of missing data points in standard data-dependent acquisition (DDA) proteomic strategies (26, 27). This analytical platform included a software for label-free quantification of the peptide ion intensities recorded in the MS data collected with DDA scanning. The MS1 signal for a given peptide is integrated for each peptide across all samples being compared and quantification achieved by considering the area under the curve of the extracted ion chromatogram. Relative peptide quantifications can be directly compared across different samples to obtain relative peptide abundances and thus measure differences between samples.

An R script was used to automate the statistical analysis by adding the intensities of individual peptides that belong to a given protein. We then compared the mean abundances between biotinylated and control labelled samples, which are cells treated with vehicle (instead of the respective biotin reagent) and represented proteins bound un-specifically to the magnetic streptavidin beads. Gene Ontology enrichment analysis (GOEA) was conducted on proteins differentially expressed between Biotin labelled *versus* Control labelled samples. The UniProtKB database (http://www.uniprot.org/) was used to access the functional information (CC, BP, MF) for GO enrichment analysis using enrichGO() function in ClusterProfiler (R) using Log2FC > 0.5 whilst the Benjamini-Hochberg adjusted *p*-value thresholds (indicated for each experiment in the results section and figure legends) was tailored according to the size and type experiment. In addition, an “in silico” database, which is a set of proteins found by machine learning to be likely located on the cell surface (obtained from Bausch-Fluck *et al* (25)) was used to assess the CC information for GO enrichment analysis using an in-house Protools2 (R package).

For reproducibility and statistical robustness purposes, biological and technical replicates were included for seven independent AML cell lines. The number of technical and biological replicates for each independent study are indicated for each experiment in the results section and its corresponding figure legend when appropriate, along with *p*-values and statistical tests. Briefly, statistical analysis was performed in RStudio (v1.2.5033). Pearson r was calculated in correlation analysis and Kruskal–Wallis test was used to assess significance in proteomics data. Where applicable, *p*-values were adjusted for multiple testing using Benjamini–Hochberg method.

A key and novel aspect of the proposed methodology is that it will be able to quantify cell surface proteins in absolute units (copy numbers per cell) in an untargeted manner. This will be important for target prioritization. This strategy was only employed for assessing the quantitative nature of SUCAM of 7 AML cell lines. For this strategy, normalized Log10.Normalised LFQ signals for proteins of known molarity, which are constituents of an external UP2 protein standard (sigma) were plotted against the number of molecules analysed (converted from molarity). Molarity can be converted to number of molecules using the Avogadro’s number after protein signals are normalized by their molecular weight. Preliminary experiments using the Universal Dynamic Range Standard mix (Sigma) showed that this method produces calibration curves with high correlation accuracy (R2>0.96) (Supplemental Table S2).

To determine absolute values (copy numbers) of cell surface proteins, we divide the LFQ quantitative values (sum of chromatographic peak areas of peptides belonging to a given protein) by the molecular weight of the respective protein. This allows normalizing quantitative values for differences in the number of peptides derived from proteins of different length. We then convert Log10.LFQ signals (normalised to Molecular weight) into number of protein molecules using the calibration curve constructed from the Universal Dynamic Range Standard mix.

## Results

### Overall Method Design and analytical Strategy

Surfaceomic approaches, based on biotinylation-based enrichment coupled with mass spectrometry proteomics, are now routine in many laboratories. However, there is a scarcity of published systematic characterization or comparative analysis of the performance of such techniques. Paramount to surfaceomic methods is that an effective enrichment requires specific isolation of proteins on the cell surface whilst minimising non-specific biotinylation, enabling a precise assessment of the surfaceome.

**Fig. 1:**
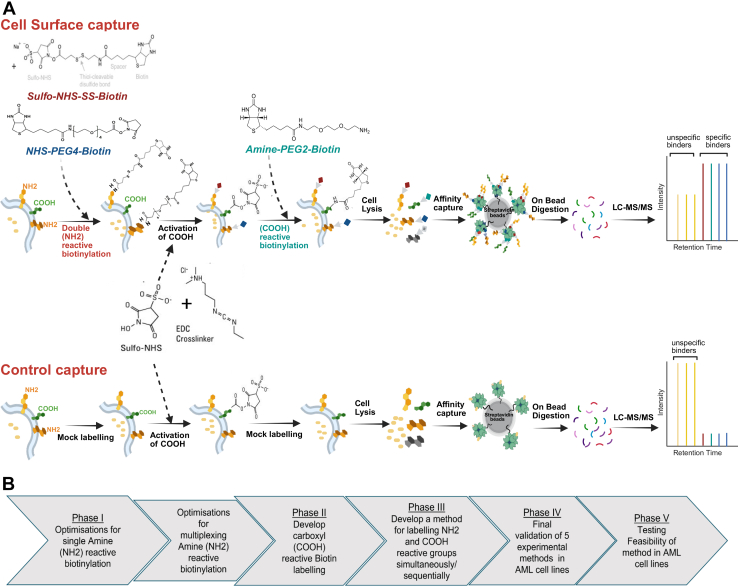
**Design and developmental overview of Surfaceome Capture by Multiplex (SUCAM) biotinylation for surfaceome exploration**. *A*, downstream workflow for cell surface proteomics. For labeling amine and carboxyl reactive groups in-combination and/sequentially, 1.2 million cells are biotinylated with two different amine reactive biotin derivatives, followed by sequential activation of carboxyl groups using catalysts/low pH and subsequently labeled with PEG2 carboxyl biotin derivative in PBS pH 8.0. Control labeling is performed on the same number of cells following the same procedure except biotin is replaced by the vehicle. Cells are lysed, biotinylated proteins enriched, digested on beads, and resulting peptides are analyzed by mass spectrometry to identify specific and nonspecific binders to streptavidin magnetic beads. *B*, workflow for developing a standardized surface protein capture quantification method is divided into five phases. Phase I includes developing a procedure for amine reactive biotinylation, validating and comparing the efficacy of two amine reactive biotin agents and developing a “multiple” amine reactive biotinylation strategy. Phase II involves developing and validating carboxyl reactive biotinylation by comparing with single amine reactive biotin agents under different conditions. Phase III involves developing a novel “multiplexing” biotinylation strategy that uses a formulation of two different amine reactive biotin derivatives with differing physio-chemical properties followed by sequential labeling with carboxyl reactive biotinylating agents. Phase IV involves a final validation and comparison of five cross-linking biotinylation strategies followed by phase V, testing feasibility of final method SUCAM in 7 AML cell lines.

Analytical factors such as cell density, nature of functional groups in reagents and their concentration and washing conditions contribute to the performance of affinity chromatography and thus on the efficacy of surfaceomic methods based on biotinylation enrichment. Thus, in this work, we aimed to advance existing cell-surface protein enrichment methods by developing a biotin derivatisation strategy based on multiple-functional group labelling coupled with MS-based proteomics. The proposed approach – named Surfaceome Capture by Multiplex (SUCAM) biotinylation, schematically shown in Figure 1*A* consists of simultaneous and/or sequential labelling of protein functional groups with biotin derivatives, with the aim of circumventing some of the existing limitations of techniques based on single biotin derivatisation of the cell surface proteome.

### Optimization of a Standardised Multiplex Surfaceomic Method

A workflow for the development, optimisation and evaluation of SUCAM is depicted in Figure 1*B*. For each step-by-step developmental stage, we evaluated the efficacy of different biotinylating reagents, both separately and in combination at capturing proteins annotated with cell surface associated ontologies relative to control pull-downs.

### Impact of cell Density on Surfaceome Enrichment

**Fig. 2:**
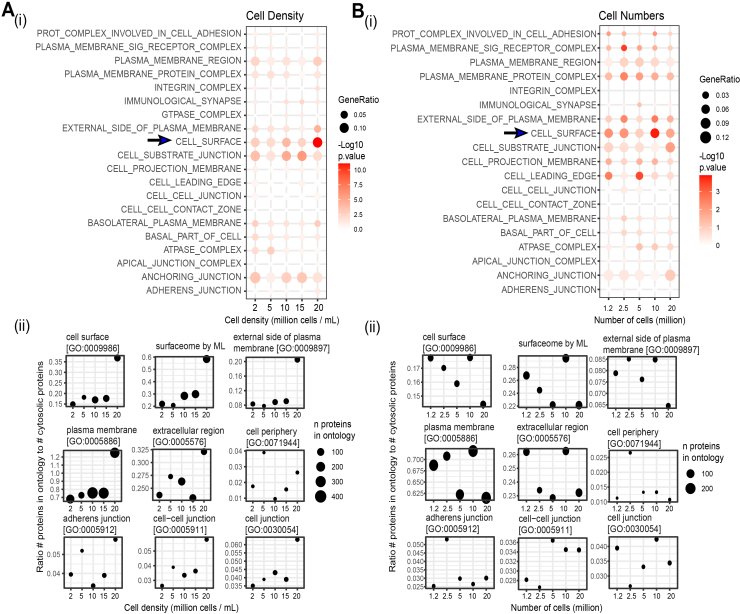
**Impact of cell density and cell numbers on the performance of amine labeling for surfaceome enrichment**. *A*, GO Enrichment Analysis (ORA) of cellular component (i) and ratio to cytoplasmic proteins (ii) for cell surface labeling of a pool of AML suspension cultures seeded at the densities shown (N = 2) in a volume of 1 ml show 20 million cells/ml to be superior at enriching for the cell surface and isolating the highest number of proteins for the cell surface ontology. *B*, GO enrichment (i) and ratio to cytoplasmic proteins (ii) for biotinylation carried out using 1.2, 2.5, 5, and 20 x10^6^cells at a density of 20 x10^6^cells/ml returns a superior enrichment and isolation of highest number of proteins for 10 million cells, followed closely by 1.2 and 2.5 million. Ontologies were obtained from Uniprot and from Bausch-Fluck *et al* (27) (Surfaceome by ML). GO enrichment analysis was performed using the clusterProfiler R package on differentially expressed proteins (DEPs) in a biotin versus control comparison (Log_2_FC > 0.5 and p-adjusted value threshold of 0.1). In panel (i), the size of the dot plots represents GeneRatio while panel for (ii) the size of the dotplot represent number of proteins for each GO term. N = 2 independent replicates were conducted for each condition, and each sample was injected twice (x2) into the system for MS.

We initially aimed to investigate how cell densities impact assay performance and nature of the isolated surfaceome. N-Hydroxysuccinimide (NHS) ester (Sulfo-NHS-SS-Biotin), employed for this optimisation were chosen as useful labelling reagents due to their relatively fast labelling reaction under physiological conditions, and have been employed before in several studies (28). Labelling of cell surface proteins was carried out at densities ranging from two to 20x10^6^cells/ml (Fig. 2). Two independent replicates were conducted for each condition, and each sample was injected twice (x2) into the system for MS. We found that a cell density of 20 million cells/ml resulted in optimal labelling and enhanced the efficacy of cell surface and membrane protein enrichment. This is evidenced in the greater enrichment of the cell surface ontology using GO Enrichment Analysis (Fig. 2*A*, panel i), as well increased ratio in the number of cell surface-associated proteins relative to cytoplasmic proteins (Fig. 2*A*, panel ii). For a cell density of 20 cells/ml, the ontologies related to “External side of plasma membrane” and “cell surface” were two out of five top-enriched GO terms.

### Impact of Cell Numbers on Surfaceome Enrichment

Having stablished that a cell density of 20x10^6^cells/ml is optimal for the isolation of cell surface protein by biotinylation, we asked whether it would be possible to increase the sensitivity of the assay by lowering the number of labelled cells but maintaining their density. This is a critical question in order to develop a sensitive assay applicable to real world clinical samples where tissue availability is limited. We thus assessed the efficacy of membrane proteome enrichment at different volumes of cell suspension, ranging from 1 ml to 0.06 ml whist maintaining the optimum density, *i*.*e*.*,* 20 x 10^6^cells/ml (Fig. 2*B*). Two independent replicates were conducted for each condition, and each sample was injected twice (x2) into the system for MS. Cell surface and membrane proteome analysis on 0.06 ml cell suspension at 20 x 10^6^cells/ml cell density, corresponding to 1.2 x 10^6^cells, produced an approximately similar cell surface to cytoplasmic ratio as the proteome analysis based on a 2, five and 10 x 10^6^cells (Fig. 2*B*, panel ii). We therefore concluded that 1.2 x 10^6^cells at a density of 20 x 10^6^cells/ml provide adequate conditions, with the added advantage of being able to scale down the assay to a manageable number of cells, useful for the analysis of primary material.

### Efficiency of cell Surfaceome Enrichment by Different Amine-reactive Biotinylating Reagents

**Fig. 3:**
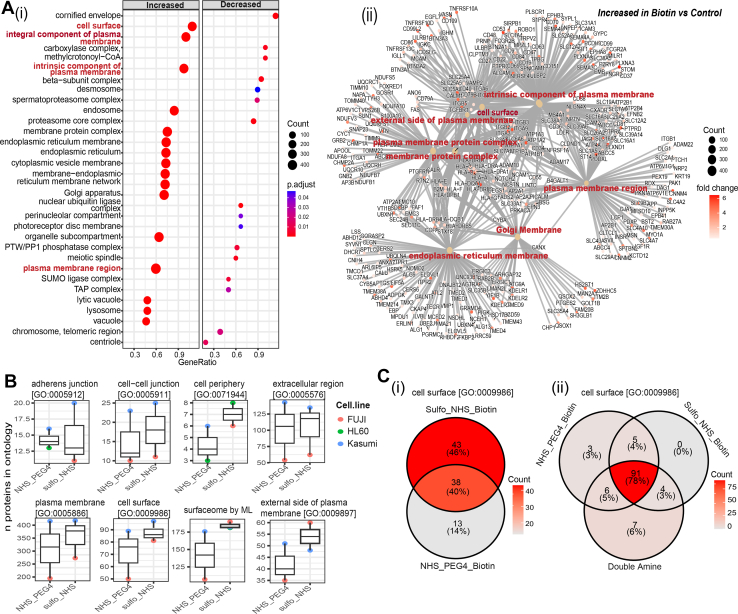
**Evaluating the efficiency of cell surface enrichment for two amine-reactive biotin conjugates both individually and in-combination**. *A*, (i) a dot plot and (ii) network visualization of GO Enrichment analysis (GSEA) performed using Gene Ontology (GO) sets restricted to cellular component (CC) depict the specificity and sensitivity of amine-reactive biotinylation reagent (Sulfo-NHS-SS) for enriching the cell surface and other CCs in p31/Fuji cell line from 20 million cells/ml starting material. Gene set enrichment analysis (GSEA) was performed using the clusterProfiler R package on differentially expressed proteins (DEPs) in a biotin versus control comparison (p-adjusted value threshold of 0.1). For (i), dots size represents the number of proteins for each GO term; the Gene ratio represents the proportion of input proteins that are associated with a specific GO term and color scale represents the enrichment *p*-value. N = 2 biological repeats were conducted for each condition, and each sample was injected twice (x2) into the system for MS. *B*, number of proteins isolated by Sulfo-NHS-SS and NHS-PEG4 in each of the named ontologies and from Bausch-Fluck *et al* (27) (Surfaceome by ML) across three AML cell lines is higher for Sulfo-NHS biotin. *C*, overlap of cell surface proteins captured by Sulfo-NHS-SS or NHS-PEG4 biotin for p31/Fuji show a highest percentage (46%) of unique proteins captured by Sulfo-NHS and 14% of the unique proteins captured NHS_PEG4. Overlap of cell surface proteins captured by Sulfo-NHS-SS, NHS-PEG4 biotin or double labelling shows the highest percentage (6%) of unique proteins captured by Double amine. For each cell line, p31/Fuji, Kasumi, HL60, N = 2 independent replicates were conducted for each condition, and each sample was injected twice (x2) into the system for MS.

GO Enrichment analysis showed that despite the efficient enrichment of the cell surface at 20 x 10^6^cells, several unspecific intracellular proteins were detected and identified in samples biotinylated with Sulfo-N-Hydroxysuccinimide (NHS) ester Sulfo-NHS-SS-Biotin (Fig. 3*A*, panel i & ii), irrespective of the analytical method applied. Although non-specific interactions of some proteins with streptavidin-beads, resistant to the stringent washings performed cannot be ruled out, control experiments performed omitting the biotinylation step indicate that these interactions alone cannot account for the detection of many intracellular proteins in the biotinylated samples. Other possible explanations can be considered: (i) intracellular proteins are released by damaged cells prior or during cell surface biotinylation and can be labeled during the biotinylation reaction; (ii) despite being negatively charged, the biotinylation reagent can penetrate into the cells either by diffusion or by means of a specific transporter (as for instance the sodium dependent multivitamin transport system (8); (iii) cytoplasmic proteins are copurified by virtue of a strong interaction with membrane and cytoskeletal proteins, which hinder identification of low abundant cell surface proteins.

To investigate if different biotinylating reagents have differential labelling properties, we evaluated the reactivity of the two most common amine-reactive biotinylation reagents: NHS-PEG4-Biotin and the Sulfo-N-hydroxysuccinimide (NHS) ester Sulfo-NHS-SS-Biotin. For each cell line, p31/Fuji, Kasumi, HL60, N = 2 biological repeats were conducted for each method, and each sample was injected twice (x2) into the system for MS. We found that the use of the Sulfo-NHS-SS-Biotin reagent returned a larger number of cell surface proteins compared to NHS-PEG4-Biotin (Fig. 3*B*). Peptide and protein identifications for surfaceomic proteins associated with amine reactive biotinylation is represented in Supplemental Table S3. For p31/Fuji cells, despite a significant percentage (40%) of identifiable cell surface proteins over-lapping between the two agents, a large percentage (46%) of unique cell surface proteins were captured with Sulfo-NHS-Biotin, whilst the percentage of unique cell surface proteins captured with NHS-PEG4-Biotin was only 14% (Fig. 3*C*, panel i). As our aim was to increase the density of the biotin label per proteins, we reasoned that by combining the two agents, we could potentially expand the coverage of the identified cell surface proteome. To test this notion, we carried out experiments using a combination of both Sulfo-NHS-SS-Biotin and NHS-PEG4-Biotin regents. In an initial experiment, this double labelling approach returned seven unique cell surface proteins not detected by single labelling approaches, whereas just 0 and three proteins were uniquely identified when using single reagents (Fig. 3*C*, panel ii). These results suggested that a combination of biotinylating reagents would increase the repertoire of identifiable cell surface proteins.

### Efficiency of cell Surfaceome Enrichment by Amine- and carboxyl-Biotinylating Reagents

**Fig. 4:**
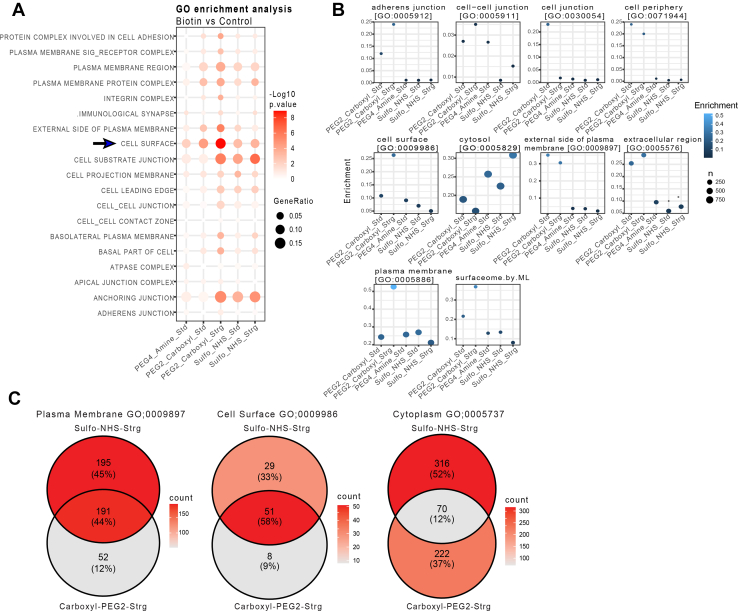
**Validation and comparison of the performance of carboxyl and single amine enrichment strategies**. *A*, For the DEPs (Biotin *versus* Control) in the Carboxyl labelling condition, GO Enrichment Analysis (ORA) showed a superior efficiency for the enrichment of the cell surface compared to amine reactive labelling with NHS-SS-Biotin and NHS-PEG4. Stringent (Strg) immunoaffinity purification conditions used to isolate carboxyl-labelled cell surface proteins significantly enhanced the enrichment of plasma membrane (PM) and extracellular, membrane-associated proteins compared to standard (Std) conditions. GO Enrichment analysis (ORA) was performed using the clusterProfiler R package on differentially expressed proteins (DEPs) in a Biotin versus Control comparison (Log_2_FC > 0.5 and p-adjusted value of 0.1 to define the thresholds). For the dot plot, dots size represents the Gene ratio, the proportion of input proteins that are associated with a specific GO term (degree of enrichment) and colour scale represents the -log10.*p*-value (statistical significance). *B*, The cell surface to cytoplasm enrichment ratio is higher for carboxyl labelling than that of amine labelling and is further enhanced using stringent affinity purification conditions. *C*, Overlap of cell surface and cytoplasmic proteins for NH2-labelling and COOH-labelling shows some unique cell surface proteins for each labelling method, with less cytoplasmic contamination by carboxyl labelling compared to amine labelling. Ontologies were obtained from Uniprot and from Bausch-Fluck *et al* (27). N = 2 independent replicates were conducted for each condition, and each sample was injected twice (x2) into the system for MS. Strg = Stringent, Std = Standard.

The observation that different biotinylating reagents return different repertoire of cell surface proteins led us to propose that labelling additional protein reactive groups would increase this coverage further. We thus tested a carboxyl-reactive biotinylation strategy as an orthogonal approach for amine-reactive biotinylation (Fig. 4). Furthermore, we also tested whether more stringent washing of beads with 2M NaCl after labelling would eliminate non-specific proteins adsorbed to the beads. When comparing the efficacy of carboxyl, Sulfo-NHS-SS-Biotin and NHS-PEG4-Biotin at isolating cell surface associated terms, we observed that carboxyl labelling was more efficient at isolating the cell surface than Sulfo-NHS-SS-Biotin (as judged by GO Enrichment analysis, Fig. 4*A*), and there was an observable improvement in isolation of the cell surface using stringent immunoaffinity conditions. Consistent with these data, the enrichment of cell-surface associated GO terms (*i*.*e*.*,* Cell periphery, Cell Surface, Plasma Membrane and Surfaceome) was highest for carboxyl labelling using stringent (Strg) affinity purifications conditions and a lower enrichment was observed for amine labelling (Fig. 4*B*, Supplementary S4A). In addition, the enrichment of proteins within the cytosolic GO Term for biotin-based reagent *versus* control/vehicle was evaluated for the different biotin labelling strategies, with evidently lower cytoplasmic contamination for Carboxyl labelling using stringent affinity purifications conditions. Peptide and protein identifications for cell surface proteomics associated with amine reactive *versus* carboxyl reactive biotinylation is represented in Supplemental Table S4, *B*–*F*.

### Carboxyl and Amine labelling Capture Different Surfaceomes

To test if amine and carboxyl labelling methods captured different sets of surfaceome-proteomes, we compared the nature and uniqueness of proteins identified with each technique. As Figure 4*C* shows, 45% of identifiable plasma membrane [GO:0009897] proteins were unique to Sulfo-NHS-SS-Biotin and 12% unique to the Carboxyl labelling, with an overlap of 44% proteins captured by both amine and carboxyl labelling. The percentage of cytoplasmic proteins unique to Sulfo-NHS-SS-Biotin was 52%, whilst contamination with cytoplasmic proteins was significantly lower for carboxyl labelling. This suggests that a combination of chemically distinct cross-linker reactive biotin agents may enable a more efficient isolation of cell surface proteins.

### Development of a Surfaceome Enrichment Method based on Sequential carboxyl and Amine labelling Capture

Spurred by the finding that a combination of biotinylating reagents isolated a unique repertoire of proteins, coupled with differing efficiencies for cell surface enrichment, we reasoned that adding a combination of double amine and carboxyl labelling compound would increase the coverage of the identified surfaceome further. The logic for this approach is that increasing the variety of functional groups that are labelled on the cell surface would increase the density of labels per protein, increasing cell surface enrichment whilst widening the physiochemical properties of the identified proteins.

**Fig. 5:**
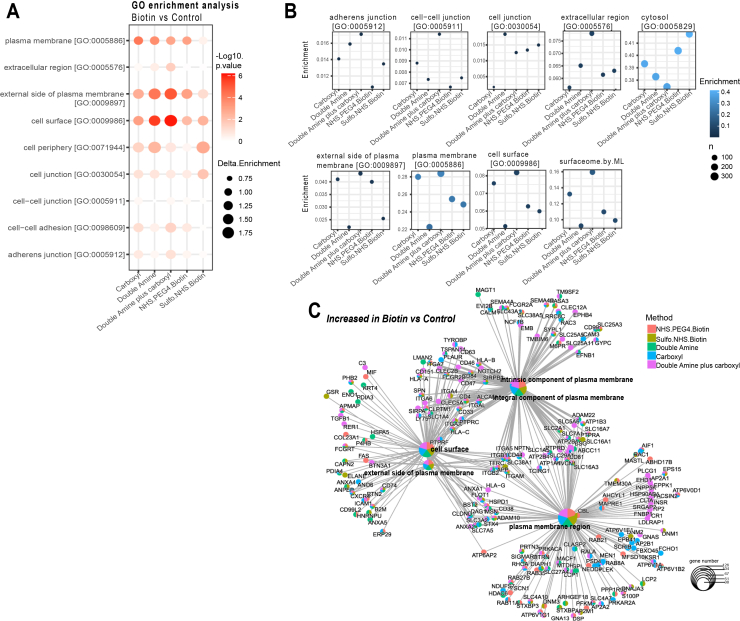
**Validation and comparison of SUCAM and other biotin conjugation methods in a pool of 4 AML cell lines**. *A*, GO Enrichment analysis restricted to cellular component (CC) showed superior enrichment of PM-associated proteome for double amine plus carboxyl method compared to other methods in a pool of 4 AML cell lines (p31/Fuji, Kasumi, HL60, NB4). The biological sub-cellular localisations of the DEPs (Biotin *versus* Control) were explored with Cellular (CC) component GO term enrichment analysis using “Protools2” R package (Log_2_FC > 0.5 and q-value <0.25). Ontologies were obtained from Uniprot and from Bausch-Fluck *et al* (27). Dot size represents Delta Enrichment, *i.e.* the proportion of biotin labelled proteins relative to the unlabelled proteins for each specific GO term. Colour scale represents -log10(*p*-value). *B*, Enrichment efficiencies for proteins obtained by each of the named biotinylation approaches was highest for double amine plus carboxyl. Ontologies were obtained from Uniprot and from Bausch-Fluck *et al* (27). *C*, A network plot visualisation for GO Enrichment analysis identifies proteins increased in Biotin labelled *versus* control labelled control (Log2FC > 0.5, *p*-value <0.25) samples in a pool of 4 AML cell lines (P31/Fuji, Kasumi, HL60-NB4) for five biotin cross-linking conditions. The network was constructed with Gene-Concept network (cnetplot) using ClusterProfiler, where small nodes represent genes and large nodes represent enriched GO terms. The GO nodes and Gene nodes are colour coded to represent the method. The size of the GO nodes represent the number of proteins identified for each of the five methods (colour coded). N = 6 independent replicates were conducted for each method, and each sample was injected twice (x2) into the system for MS.

To test the validity of these assumptions, we performed a final comparison of the performance of five different cell surface biotinylation methods using single amine reactive biotin derivatives, combination of two amine-reactive conjugates, carboxyl reactive conjugation and a newly developed “multiplexed” procedure combining a formulation of double amine and carboxyl reactive reagents in a single reaction. The latter approach was named SUCAM (Surfaceome Capture by Multiplex Biotinylation). These modified cell surface labelling strategies were compared using a reduced quantity of starting material (1.2 x 10^6^cells per reaction) separately for seven cell lines (data not shown) and a pool of 4 AML (P31/FUJ, HL60, NB4 and Kasumi) cell lines (Fig. 5). For the experiment performed on a pool of 4 AML cell lines (P31/FUJ, HL60, NB4 and Kasumi), N = 6 independent biological replicates were conducted for each method, and each sample was injected twice (x2) into the system for MS.

For pool AML cells, consisting of P31-FUJ, HL60, NB4 and Kasumi, SUCAM returned a greater significance of enrichment for “Cell Surface GO;0009986” proteins (*p*-value <8.57E-07) than the other methods tested (Fig. 5*A*, Supplemental Table S5*A*). Consistently, the enrichment of proteins with GO ontologies associated to the cell surface (Cell periphery, Cell Surface, Plasma Membrane and Surfaceome) for the experimental biotin-based reagent *versus* control/vehicle was also found to be higher for labelling strategies using either just double amine or SUCAM (*i*.*e*.*,* double amine plus carboxyl) (Fig. 5*B*, Supplemental Table S4*B*). Coincidently, the enrichment of proteins within the cytosolic GO Term for biotin-based reagent *versus* control/vehicle was found to be lower for labelling strategies using SUCAM (Fig. 5*B*).

A network plot in GO Enrichment Analysis, (Fig. 5*C*), shows the relationship between PM-associated GO terms enriched in Biotin *versus* Control across five biotin conjugation methods. An analysis of how different GO terms are interconnected across the five conjugation methods shows that SUCAM has a superior efficacy at enriching for the intersections between functional modules annotated as plasma membrane-associated, *i*.*e*.*,* the proteome shared by the GO terms Cell Surface, Intrinsic component of plasma membrane, external side of plasma membrane and/or the Plasma Membrane region. SUCAM thereby identifies these PM-associated GO terms as the most important and functionally interconnected modules.

**Fig. 6:**
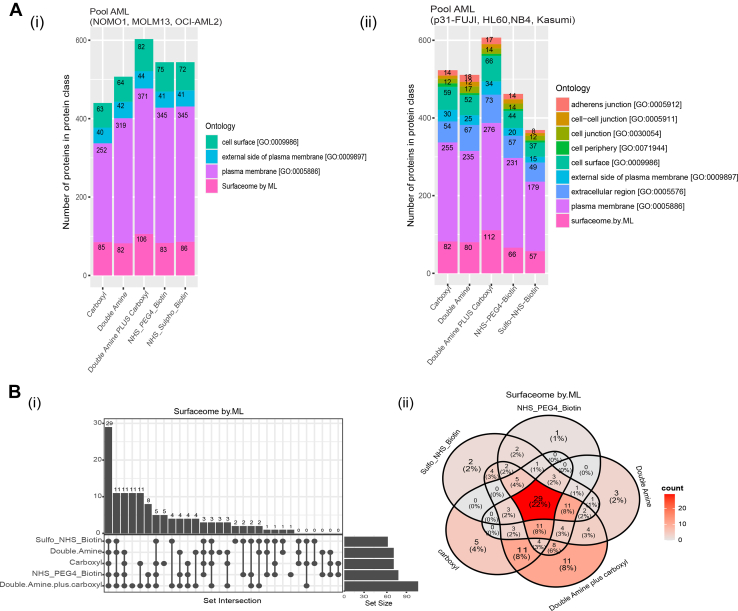
**Absolute numbers and overlaps of the cell surface associated proteome obtained by SUCAM and other biotin conjugation strategies**. *A*, (i) Total number of DEPs (Biotin *versus* Control) proteins identified by the named methods are shown in a pool of 3 AML cell lines (NOMO1, MOLM13, OCI-AML2) relative to control. (ii) Total number of DEPs (Biotin *versus* Control) proteins identified by the named methods are shown in a pool of 4 AML cell lines (p31/Fuji, Kasumi, NB4, HL60) relative to control. The proposed SUCAM (a.k.a. double amine plus carboxyl) approach was superior to other methods for detection of quantifiable proteins (Log_2_FC > 0.5, *p*< 0.25 were considered statistically significant) annotated to be present in a wider cohort of cell surface-associated ontologies. Ontologies were obtained from Uniprot and from Bausch-Fluck *et al* (27) (Surfaceome by ML). *B*, (i) Upset plot visualising the set size and intersections of DEPs (Biotin *versus* Control) for the five different biotin methods in a pool of 3 AML cell lines (NOMO1, MOLM13, OCI-AML2) shows the highest absolute number of proteins present in Surfaceome by ML was captured by double amine plus carboxyl. The highest number of unique proteins (n = 11) was captured by Double amine plus carboxyl. (ii) Percentages and overlaps of cell surface proteins isolated by the named methods and present in the Surfaceome by ML reflects that the highest percentage (8%) of unique proteins captured by SUCAM. For each pool of AML cell line suspensions, N = 2 independent replicates and X2 MS technical repeats (injections) were conducted for each method.

The absolute number of proteins in cell surface-associated ontologies was higher for double amine plus carboxyl (Fig. 6*A*, Supplemental Table S5*C*) for (i) a pool of 3 AML cell lines consisting of NOMO1, MOLM13 and OCI-AML2 and (ii) a pool of 4 AML cell lines consisting of p31-/Fuji, Kasumi, HL60, NB4). As an example, the absolute number of proteins annotated as Surfaceome by ML was higher for double amine plus carboxyl for a pool of 4 AML cell lines consisting of p31/Fuji, Kasumi, HL60, NB4 (Fig. 6*B*, panel i). For this study, N = 6 independent biological replicates were conducted for each method, and each sample was injected twice (x2) into the system for MS. In addition, when assessing the numbers and overlaps of cell surface proteins across the tested methods, we found just 22% overlap of all cell surface associated proteins across all the five methods, with the highest percentage (8%) of unique proteins captured by SUCAM for Surfaceome by ML ontology (Fig. 6*B*, panel ii, Supplemental Table S5*D*). Peptide and protein identifications for cell surface proteomics associated with biotin cross-linking method comparison is highlighted in Supplemental Table S6.

Further analysis of all plasma membrane-associated GO terms for p31-Fuji cell line returns the highest absolute number of proteins isolated with SUCAM compared to the other biotin cross-linking methods (Supplemental Fig. S1*A*, panel i, Supplemental Table S7*A*). Assessing the proteome set size across ALL plasma membrane associated GO ontology terms led to the identification of 811 proteins enriched with SUCAM (Supplemental Fig. S1*B*, panel i, Supplemental Table S7*B*), with the highest percentage (6%) of unique proteins captured by SUCAM for all GO ontologies combined (Supplemental Fig. S1*B*, panel ii, Supplemental Table S7*B*).

Pearson correlation analysis was used to evaluate the association between the five biotin conjugation methods (Supplemental Fig. S2) in a pool of 4 AML cell lines (p31-/Fuji, Kasumi, HL60, NB4). N = 6 independent biological replicates were conducted for each method, and each sample was injected twice (x2) into the system for MS. A histogram of Pearson correlation coefficients showing the distribution of a single method Supplemental Fig. S2*A*) and a correlation plot (Supplemental Fig. S2*A*) or correlogram (Supplemental Fig. S2*A*) for pairwise correlations between multiple biotin conjugation methods show a weak correlation between double amine plus carboxyl and carboxyl alone (negative correlation coefficient (r) value, −0.024), whilst a stronger correlation was observed between Double Amine plus carboxyl and Double Amine, as shown by a positive correlation coefficient (r) value (0.37). A moderately weak correlation was observed between Double Amine plus carboxyl and NHS-PEG4 and Sulfo-NHS (single Amines), (as shown by low correlation coefficients, 0.19 and 0.20 respectively).

For assessing the efficiency of cell surface biotinylation with SUCAM, a scatterplot showing the relationship between the abundance of the surfaceome (log_2_ fold change, biotin *versus* control) and the Log10.absolute quantifications of the total proteome from total cell lysates was used to assess for overlapping proteins (Supplemental Fig. S3*A*). 20% of biotinylated cell surface proteins, that is, cell surface proteins that are significantly increased in biotin *versus* control overlap with the quantifiable proteome from the total cell lysate, highlighting the significant enrichment of the surfaceome. In addition, surfaceomic proteins increased in biotin *versus* control represent 47% of the total biotinylated proteome expression (Log_2_FC > |0.5| for proteome and surfaceome data) for SUCAM (Supplemental Fig. S3*B*), indicating high selectivity and sensitivity of SUCAM for enriching of the surfaceome.

To complement these data, we performed western blotting analysis of a cell surface proteins (CD31 and CD38) and compared it to a cytoplasmic protein (Annexin V and HSP90) to confirm the membrane-impermeability of the biotin reagent and the specificity in the localisation of the cell surface protein isolated by SUCAM. The specificity was confirmed as the cell surface proteins, CD31 and CD38 are present only in the biotinylated surface fraction of proteins eluted from Streptavidin matrix and not in the unbound (unlabelled) fraction. The cytoplasmic proteins, Annexin V and HSP90 are mostly present in the unbound fraction (Supplemental Fig. S6, Supplemental Table S7*C*). Therefore, the data is consistent with the enrichment analysis of surfaceomic data suggesting that SUCAM specifically isolates cell surface proteins rather than intracellular proteins from AML cell lines.

### SUCAM Produces the Highest Yield of the Surfaceome out of all Methods Tested

**Fig. 7:**
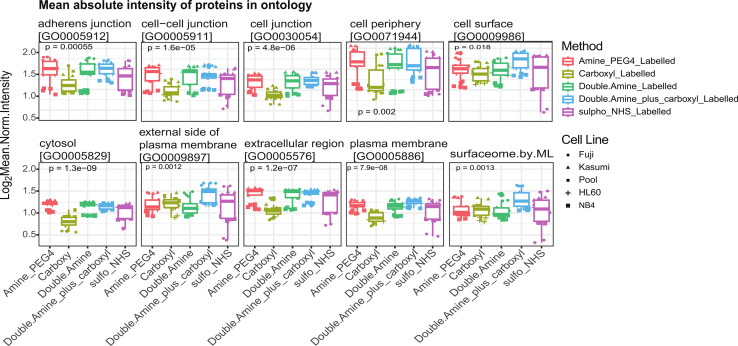
**Evaluation of cell surface-associated protein abundances across five biotin conjugation methods**. Mean normalised protein abundances (measured as the sum of areas from XICs of the different peptides that belong to a given protein) for proteins associated to the named ontologies are shown for SUCAM (a.k.a. double amine plus carboxyl) and other biotinylation methods four AML cell lines. Ontologies were obtained from Uniprot and from Bausch-Fluck *et al* (27). Significance was determined by Kruskal-Wallis test. Dots represent median log_2_-transformed intensity distributions. Lines connect medians across inputs. For each of the 4 AML cell lines, N = 2 independent replicates, and X2 MS technical repeats (injections) were conducted for each method.

So far, we evaluated the performance of the different methods by counting how many proteins annotated with ontologies related to cell surface localization were identified by each of the labelling strategies. As an additional readout to assess the efficacy of the different labelling methods tested, we also evaluated the abundances of proteins that belong to each such subcellular location (measured as the sum of areas from XICs of the different peptides that belong to a given protein). We found that the mean normalised abundances of proteins annotated to a number of cell surface-associated GO terms including proteins annotated to the Cell Surface GO;0009986, External side of plasma membrane GO;0009897 and surfaceome by ML were significantly higher (*p* = 0.018, *p* = 0.0012 and *p* = 0.0013, respectively by Kruskal-Wallis test) across four AML cell lines for SUCAM (Fig. 7, Supplemental Table S8). For each cell line, N = 2 independent biological replicates were conducted for each method, and each sample was injected twice (x2) into the system for MS.

Further support for the superiority of this method is shown in Supplemental Figure S4*A*, which depicts z-score normalised heatmap for POOL AML. Similarly, Supplemental Figure S4*B* shows higher intensity of cell surface-associated proteins isolated by SUCAM in a POOL of 4 AML cell lines (P31/Fuji, Kasumi, NB4, HL60) and separately, in P31/Fuji and HL60 cell lines when compared to the other biotin cross-linking methods, with evidently higher expression and a wider coverage of cell surface proteins.

### Assessing Qualitative and Quantitative Nature of SUCAM Across AML Cell Lines

**Fig. 8:**
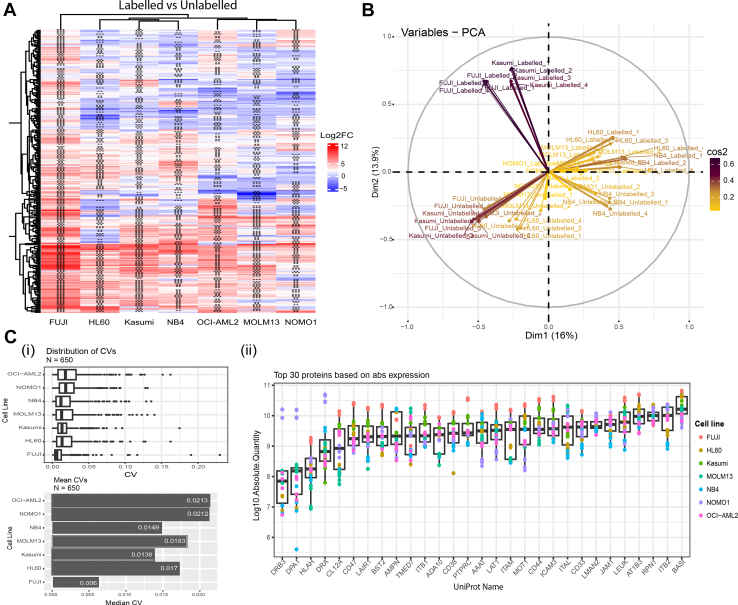
**Evaluating the quantitative nature of SUCAM across AML cell lines**. *A*, Heatmap representing the Log_2_FC values of differentially expressed cell surface proteins in Biotin labelled *versus* Control unlabelled conditions for seven AML cell lines. Based on the "Limma" package, empirical Bayesian method was used to screen for significantly different surfaceomic proteins, significance thresholds; *p*< 0.25 (∗), *p*< 0.1 (∗∗), *p*< 0.05 (∗∗∗∗). *B*, A principal component analysis (PCA) plot showing clustering of seven AML cell lines based on their cell surface protein expression levels in “biotin-labelled” and “unlabelled” datasets. The clustering suggests similarities and differences in the surfaceomic profiles of seven AML cell lines and the separation of the "biotin-labelled" and "unlabelled" datasets implies that the labelling process has created a systematic difference in the data, likely due to the biotinylation technique itself. *C*, (i) Distribution and median Coefficient of Variation (CV’s) for plasma membrane associated proteins (PMP) across replicates for individual cell lines. (ii) Absolute quantification (Log10.Absolute.Quantification) for top 30 cell surface proteins. Absolute Quantification is determined by converting Log10.LFQ signals (normalised to Molecular weight) into number of protein molecules using the calibration curve constructed from the Universal Dynamic Range Standard mix. N = 4 independent replicates for the P31/Fuj, HL-60, MOLM13, NOMO1, and NB4 and N = 2 independent replicates for Kasumi-1 and OCI-AML2, and X2 MS technical repeats (injections) were conducted for each cell line.

A key and novel aspect of the proposed methodology is that it will be able to quantify cell surface proteins in absolute units (copy numbers per cell) in an untargeted manner. This will be important for target prioritization. To investigate the applicability of the final SUCAM method, we applied it to the detection and quantification in absolute units of cell surface proteome in seven AML cell lines. Absolute Quantifications of the cell surface proteome for 7AML cell lines highlighted in Supplemental Table S9*A*. Log2 transformed absolute quantifications (Supplemental Table S9*B*) have been used for differential expression analysis using “Limma” package and the Bayesian method to screen for significantly increased proteins in Biotin labelled *versus* unlabelled cell surface. This was represented as a Log_2_FC heatmap obtained from Log2 transformed absolute quantifications of the surfaceome (Fig. 8*A*, Supplemental Table S9*C*), from which we identified 434 proteins with ontologies relating to the surfaceome across the seven AML cell lines. A principal component analysis (PCA) generated from expression levels (z scores of median Log2.Absolute Quantification of the Surfaceomic proteins) shows distinct clustering of seven AML cell lines based on their cell surface protein expression levels suggesting similarities and differences in the surfaceomic profiles of seven AML cell lines, outlining disease heterogeneity. The separation of the "biotin-labelled" and "unlabelled" datasets implies that the labelling process has created a systematic difference in the data, likely due to the biotinylation technique itself, (Fig. 8*B*, Supplemental Table S9*D*).

For this study, four independent replicates for the P31/Fuj, HL-60, MOLM13, NOMO1, and NB4 and two independent replicates for Kasumi-1 and OCI-AML2, and X2 MS technical repeats (injections) were conducted for each cell line. To examine its quantitative nature, SUCAM experiments were carried out in biological replicates (four independent replicates for the P31/Fuj, HL-60, MOLM13, NOMO1, and NB4 and duplicates for Kasumi-1 and OCI-AML2). Assessment of replicates allowed us to calculate the reproducibility (precision) of quantitative method, which we evaluated by calculating coefficient of variations (CVs, *i*.*e*.*,* standard deviation divided by mean) for individual proteins across replicates. We found that median CVs were below 2.5% for all cell lines analysed, with an overall median precision of 1.7% (range between 1% and 2.5%, Fig. 8*C*, panel i). The variables in this study include all surfaceomic proteins, N = 650.

Absolute quantification is necessary to explore the nature of the most abundant cell surface proteins in these cell models. The top 30 more abundant proteins by Log10.Absolute quantities in these models, shown in (Fig. 8*C*, panel ii, Supplemental Table S9*F*), include well known cell surface proteins in AML – such as CD43 (LEUK), CD50 (ICAM3), CD44, CD33 – but also other less well-known polypeptides with potential unexplored roles in this disease, including BASI (CD147, Basigin), AT1B3 (CD298) and RPN1 (Ribophorin-1). Supplemental Table S10 includes peptide and protein identification data associated with the cell surface proteomics characterised by SUCAM for 7 AML cell lines. Overall, these data show that SUCAM enriches and quantifies the surfaceome with quantitative precision and with a breath that allows discovering potential new drug targets.

Functional Enrichment analysis allows for identification of molecular pathways and biological processes associated with biotinylated surface-omic proteins isolated and detected with SUCAM in a pool of 4 AML cell lines (P31/Fuji, Kasumi, HL60, NB4). A multi-group GSEA analysis which includes the enrichment of GO terms across biological process (BP) and molecular function (MF) categories was conducted to fully characterise Surface-omic proteins isolated by SUCAM (Supplemental Fig. S5 & Supplemental Table S11). In Supplemental Fig. S5, Z-score normalisation of differentially expressed proteins (DEPs) provides a standardized measure of how enriched or depleted a specific Gene Ontology (GO) term is in the biotin-treated group compared to the control across biological processes (BP) and molecular function (MF) GO: categories. By using the z-score to indicate the direction of enrichment (up or down-regulated) alongside the -Log10.*p*-value for statistical significance we can provide a more robust ranking of enriched GO terms. Interrogating the outcomes of multiple enrichment testing reliably identifies functionally and biologically interpretable subsets of the proteome significantly enriched using the SUCAM method. In the BP category, these terms are predominantly related to the immune response, cell proliferation, adhesion, cell-cell signalling, myeloid leukocyte activation and regulation of intracellular signal transduction. There are well-established mechanisms involved in cancer and complex diseases. The MF category includes transmembrane signalling receptor activity, protein domain specific binding, cell adhesion mediator activity. This provides a biologically meaningful protein subset for downstream classification and potential biomarker and drug target discovery.

### Systematic Comparison of SUCAM and N-glycosylation Biotin Targeting Enrichment Strategies in Solid Tumour

We first optimized dissociation strategies using solid tumour derived cell line to ensure compatibility with biotin labelling and enrichment using low input cells by assessing the depth of cell surface proteome detection. The most optimum dissociation conditions at enriching and isolating the cell surface proved to be enzymatic dissociation with collagenase. We also compared surface proteome analysis in biotin labelled cells in a monolayer with biotin labelling of cells dissociated into single-cell suspensions. Direct biotinylation in a monolayer of cells labelled with with Sulfo-NHS_Biotin and NHS-PEG4-Biotin returned a lower significance of enrichment and isolated fewer proteins with cell surface or PM ontologies. To compare and benchmark SUCAM and N-glycosylation biotin targeting enrichment strategies using the same cell model, we used the hepatocellular carcinoma derived cell line (JHH4) to assess quantitative performance, sensitivity and selectivity for cell surface enrichment from a relatively low input amount (1.2x10^6^).

To assess the reproducibility of the methods, annotated PM proteins from three biological replicates per method were characterized on a qualitative and quantitative level. GO Enrichment Analysis was employed to identify statistically significant, reproducible functional modules (*i*.*e*.*,* plasma membrane localization) across the replicates, confirming whether the functional annotation of the dataset is consistent. All methods were assessed with respect to their yield of PM-associated proteins.

We first aimed to compare the performance of SUCAM strategy with single amine labelling, double amine and carboxyl labelling strategies in one assay (exp.no 1) and then separately compared SUCAM, carboxyl and Glycoprotein capture in another assay (exp.no 2) in 1.2 x 10^6^ single-cell suspensions obtained from enzymatic dissociation of HCC cell line (JHH4). Comparisons were made within-run and between-run.

**Fig. 9:**
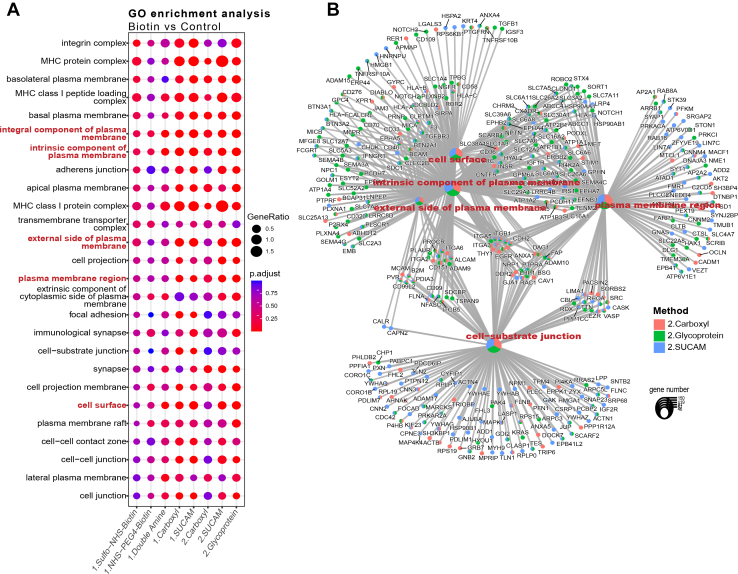
**Comparing enrichment efficiencies for SUCAM and N-glycosylation biotin targeting enrichment strategies in solid tumour**. Two experimental runs (labelled no. 1 & 2) compared SUCAM biotin labelling with four complementary labelling methods (NHS-PEG4-Biotin, Sulfo-NHS-Biotin, Double Amine and Carboxyl) and then separately with Glycoprotein capture, respectively on 1.2 x 10^6^ JHH4 hepatocellular carcinoma (HCC) cells. Comparisons were made within-run and between-run. A. Based on the two experimental comparisons, GO Enrichment analysis restricted to cellular component (CC) showed superior enrichment of PM-associated proteome for SUCAM and N-Glycoprotein enrichment strategies compared to other methods in single cell suspension obtained from collagenease dissociated adherent Hepatocarcinoma cell line (JHH4). The biological sub-cellular localisations of the DEPs (Biotin *versus* Control) were explored with Cellular (CC) component GO term enrichment analysis using “Protools2” R package (Log_2_FC > 0.5 and q-value <0.25). Ontologies were obtained from Uniprot and from Bausch-Fluck *et al* (27). Dot size represents the Gene ratio, *i*.*e*.*,* the proportion of input proteins, that is, biotin labelled relative to the unlabelled proteins that are associated with a specific GO term. Colour scale represents Benjamini-Hochberg (BH) adjusted *p*-value. For biotin labelling conditions run in Exp.no 1, N = 2 independent replicates were conducted for each method, and each sample was injected twice (x2) into the system for MS. For biotin labelling conditions run in Exp.no 2, N = 3 independent replicates were conducted for each method, and each sample was injected twice (x2) into the system for MS. *B*, Based on experimenatal comparison in Exp.no 2, a network plot visualisation for GO Enrichment analysis identifies proteins increased in Biotin labelled *versus* control (Log2FC > 0.5, *p*-value <0.25) samples in single-cell suspensions obtained from enzyme dissocaited JHH4 adherent cells for three biotin cross-linking conditions (SUCAM, N-Glycoprotein and Carboxyl). The network was constructed with Gene-Concept network (cnetplot) using ClusterProfiler, where small nodes represent genes and large nodes represent enriched GO terms. The GO nodes and Gene nodes are colour coded to represent the method. The size of the GO nodes represent the number of proteins identified for each of the three methods (colour coded). N = 3 independent replicates were conducted for each method, and each sample was injected twice (x2) into the system for MS.

Consistently, the enrichment of ontologies associated to the cell surface (Cell periphery, Cell Surface, Plasma Membrane region, Integral component of plasma membrane and External Side of Plasma Membrane) were found to be higher for labelling strategies using either SUCAM or N-Glycoprotein enrichment strategies compared to the other biotin-conjugation labelling strategies (Fig. 9*A*). GO Enrichment analysis demostrates that the glycoprotein with aminooxy-biotin resulted in marginally greater enrichment of cell surface proteins than SUCAM (*p*-value = 6.14 x 10^-5^ and 2.34 x 10^-5^, respectively). Both SUCAM and N-glycoprotein enrichment returned a greater enrichment efficiency for the plasma membrane region (*p*-value = 1.17 x 10^-5^ and 7.04 x 10^-3^ respectively, Fig. 9*A*). In addition, SUCAM or N-glycoprotein labelling conditions were the most superior at enriching for the external side of plasma membrane compared to the other biotin labelling complementary methods (*p*-value = 1.17 x 10^-5^ and 7.04 x 10^-4^, respectively) (Fig. 9*A*).

A network plot in GO Enrichment Analysis (Fig. 9*B*), shows the relationship between PM-associated GO terms enriched in Biotin *versus* Control across SUCAM, N-Glycoprotein and Carboxyl labelling strategies. The network shows a partial overlap in the proteins isolated by each strategy suggesting that these approaches identify different subsets of the surfaceome and are thus complementary.

**Fig. 10:**
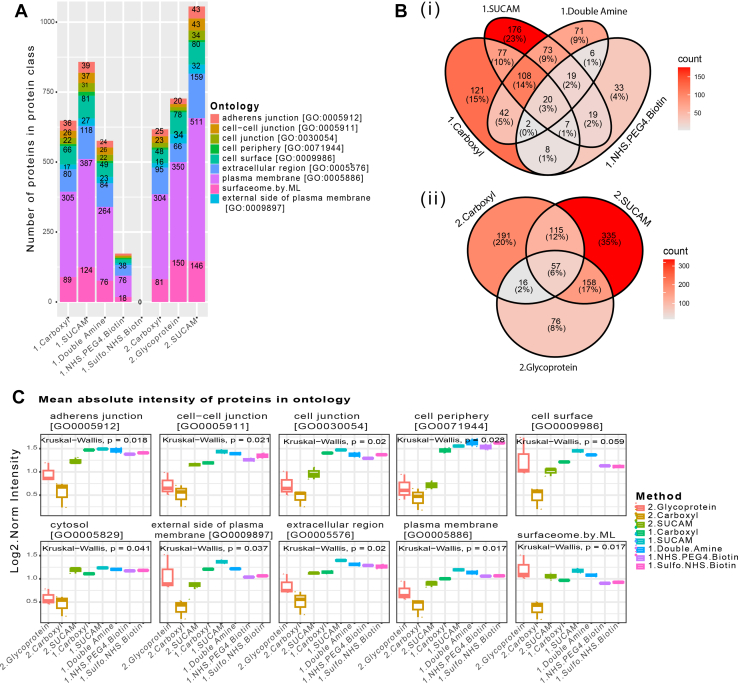
**Comparing cell surface-associated protein numbers and abundances for SUCAM and N-glycosylation biotin targeting enrichment strategies in solid tumour**. Two experimental runs (labelled no. 1 & 2) compared SUCAM biotin labelling with four complementary methods (NHS-PEG4, Sulfo-NHS, Double Amine and Carboxyl) and then separately with Glycoprotein capture, respectively on 1.2 x10^6^ JHH4 hepatocellular carcinoma (HCC) cells. Comparisons were made within-run and between-run. A. Based on the two experimental comparison (exp no.1 & 2), the total number of DEPs (Biotin *versus* Control) proteins identified by the named methods are shown in single cell suspensions of enzyme dissociated adherent cell line (JHH4) relative to control. The proposed SUCAM (a.k.a. double amine plus carboxyl) approach was superior to other methods, including Glycoprotein capture for detection of quantifiable proteins (Log_2_FC > 0.5, *p*< 0.25 were considered statistically significant) annotated to be present in a wider cohort of cell surface-associated ontologies. Ontologies were obtained from Uniprot and from Bausch-Fluck *et al* (Surfaceome by ML). *B*, (panel i and ii) Venn Diagram visualising the numbers and overlaps of DEPs (Biotin *versus* Control) in a single cell suspension obtained from JHH4 adherent cell line shows that the highest percentage of unique PM-associated proteins (n = 176 (23%), n = 335 (35%) for exp.no.1 and 2, respectively) was captured by SUCAM within all cell surface associated ontologies. *C*, Mean normalised protein abundances (measured as the sum of areas from XICs of the different peptides that belong to a given protein) for proteins associated to the named ontologies are shown for a comparison between NHS-PEG4-Biotin, Sulfo-NHS-Biotin, Double Amine, Carboxyl and SUCAM (exp. no 1) and a comparison between Glycoprotein capture, carboxyl and SUCAM labelling (exp. no 2). Ontologies were obtained from Uniprot and from Bausch-Fluck *et al*. Significance was determined by Kruskal-Wallis test. Dots represent median log_2_-transformed intensity distributions. Lines connect medians across inputs. For biotin labelling conditions run in exp.no 1, N = 2 independent replicates were conducted for each method, and each sample was injected twice (x2) into the system for MS. For biotin labelling conditions run in exp.no 2, N = 3 independent replicates were conducted for each method, and each sample was injected twice (x2) into the system for MS.

Despite aminooxy-biotin and SUCAM methods showing similar level of purity of the cell surface proteins, absolute numbers of all PM-associated proteins isolated were higher for SUCAM (Fig. 10*A*). Based on experimental comparison, SUCAM, followed by glycoprotein enrichment, scored higher in terms of sensitivity and yielded the highest absolute number of all PM-associated proteins (Fig. 10*A*, Supplemental Table S12*A*). Specifically, based on experimental comparisons, SUCAM provided a greater coverage of “plasma membrane” and “extracellular” proteins than glycoprotein enrichment and isolated the largest number of unique proteins, with 23% (exp.no 1) and 35% (exp.no.2) of cell surface proteins being unique to this method (Fig. 10*B*, panel i & ii). Furthermore, SUCAM returned the largest normalised abundances of cell surface proteins relevant to CRC pathology relative to the four other biotin-conjugation and glycoprotein enrichment strategies investigated (Fig. 10*C*, Supplemental Table S12*C*). To conclude, enzymatic dissociation of solid tumour cell line using collagenase enabled robust surfaceome profiling from 1.2 million cells. The resulting cells were amenable to SUCAM labelling and enrichment, which showed complementarity with glycoprotein enrichment, and was superior in terms of number of proteins identified. We provide all peptide and protein identifications for cell surface proteomics obtained with SUCAM and glycoprotein reactive biotinylation in Supplemental Table S13.

## Discussion

This study presents the development of a strategy to isolate the cell surface proteome using a combination of biotin-conjugates with distinct functional groups. After labelling cell surface proteins with multiple biotinylation reagents, our approach, named SUCAM, employs label free quantification of peptide ion intensities collected with DDA, as in previous studies focused on expression proteomics and phosphoproteomics (23, 24, 26, 27, 29, 30). The quantitative data is used to identify differentially abundant proteins between biotinylated and control treated samples, thus allowing us to distinguish biotinylated cell surface proteins from those that bind to beads non-specifically. The final SUCAM protocol enabled precise assessment of the surfaceome-proteome from a relatively small quantity of starting material (1.2 M cells), differing from most reported surfaceomic studies that required large cell numbers as input (typically ∼10^7^cells per experiment) (19, 31), although a recent study has screened the glycoprotein comprehensively with <1 x 10ˆ6 cells (21). Furthermore, unlike surfaceomic methods optimised for low cell numbers, which require custom and manual fabrication of packed tips (6), SUCAM employs magnetic beads, and it can therefore be used in a high throughput manner and in laboratories that do not have tip packing capabilities. Another important distinction of our study is that we determined the analytical reproducibility (precision) of the approach for the quantification of cell surface proteins. Precise quantification allows accurate ranking of the identified proteins in order of abundance, an important requirement for quantitative biology and target prioritization.

To compare the performance of methods, we first used established amine-reactive biotinylation reagent to AML cell lines; namely, Sulfo-NHS-SS-Biotin and NHS-PEG4-Biotin. In general, labelling with Sulfo-SS-NHS-Biotin resulted in a more specific, selective labelling and improved enrichment of the cell surface proteins than when NHS-PEG4-Biotin was employed. This is consistent with this reagent being preferentially used in the literature (31). The improved enrichment of the cell surface proteome with Sulfo-NHS-SS-Biotin may reflect the cleavage of the disulphide bond between protein and biotin in the reducing milieu of the cytoplasm and, as a consequence, prevent the labelling of cytoplasmic proteins. In addition, the negatively charged sulfo-NHS group prevents this reagent, at least in principle, from permeating cell membranes; therefore, Sulfo-NHS-SS was expected to biotinylate cell surface proteins only. However, cytoplasmic proteins were identified with the use of both labelling techniques. An additional difference in the chemical properties of the two biotin agents is the length of the PEGylated linker, which is longer for NHS-PEG4-Biotin and this may lead to lower steric hindrance in comparison to Sulfo-NHS-SS-Biotin. These different chemical properties of the two biotin agents may result in differences in labelling efficiency of proteins with distinct physiochemical properties and conformations. In support of this assumption, NHS-PEG4 and Sulfo-NHS-SS-Biotin identified a different repertoire of cell surface proteins, with 14% and 46% of unique proteins identified by each approach, respectively.

In an effort to extend the number of labelled and identified cell surface proteins, we combined the two biotinylating reagents in a single reaction, resulting in a wider range of proteins annotated with cell surface ontologies. Although widely used in the literature (32), and despite our observations on the usefulness of amine labelling for the isolation of the surfaceome, we reasoned that labelling the extracellular lysine side chain may have limitations, as some cell surface proteins may not be biotinylated due to lack of exposed extracellular lysine residues available for covalent labelling. We thus wanted to test the hypothesis that carboxyl labelling would provide an orthogonal means to complement the repertoire of cell surface proteins that can be identified using proteomics techniques.

Carboxyl reactive conjugation agents label surface exposed carboxyl groups on aspartic and glutamic acids, and protein C-termini. The frequencies of these residues, especially on the extra-cellular transmembrane proteins (TMPS) are larger than that of lysine residues. Indeed, 13% of the extracellular regions of these transmembrane proteins are estimated to have Asp and Glu residues, while Lys residues are reported to be 5.25% (33). Thus, carboxyl labelling can potentially isolate cell surface proteins with more efficiency than amine labelling. However, to our knowledge, carboxyl reactive conjugation has only been attempted on live cells by Ozkan Kucuk (33); otherwise, its main application is in bioconjugate chemistry of purified peptides.

Labelling of surface-exposed carboxyl groups is optimal at relatively low pH and can thus potentially compromise the integrity of the plasma membrane. This may explain why the procedure has been rarely utilised for labelling the cell surface proteome in live cells compared to amine reactive labelling, notwithstanding the recent study by Ozkan Kucuk (33). We thus wanted to revisit and evaluate the efficacy of carboxyl labelling, relative to that provided by amine-labelling reagents, for which we specifically focused on the identified plasma membrane proteins to reveal any possible method-based selectivity against a specific protein subgroup. We found that cell surface and plasma membrane-associated proteins were more enriched (relative to proteins in other subcellular compartments) with carboxyl biotinylation than with amine labelling. This finding indicates that carboxyl labelling specifically conjugated cell surface and plasma membrane proteins with minimal isolation of cytoplasmic proteins. The most significantly enriched ontologies in amine biotinylation were stable structures such as integrin complex, plasma membrane signalling receptor complexes, with evidence of more contamination with non-specific intra-cellular proteins.

We also observed that surfaceomic experiments using amine and carboxyl labelling identified unique sets of cell surface proteins with only partial overlap in identifications, indicating that labelling both amine and carboxyl groups in separate reactions would capture a broader set of proteins than either technique alone. We thus developed a formulation of labelling reagents consisting of a multiplex of biotin derivatives for multiple functional group conjugation of the surfaceome-proteome, in a strategy named SUCAM. The final protocol could detect hundreds of proteins annotated as being cell surface in ontology databases with high quantitative precision from just 1.2 x 10^6^cells.

We found that this reagent formulation, the basis of SUCAM, was superior to single amine, double amine and carboxyl conjugation, leading to the isolation and identification of a higher number of cell surface and cell surface-associated proteins. Considering the recent developments in surfaceomic workflows based on N-glycocapture from low-input cells isolated from solid tissue, we also compared the performance of SUCAM and N-Glycoprotein labelling strategy in a single-cell suspension isolated from enzymatic dissociation of HCC cell line.

Our comparative studies of cell surface protein enrichment methods, both aminooxy-biotin and SUCAM showed high level of purity and specificity for the PM-associated ontologies. However, SUCAM outperformed other approaches in terms of the absolute number of proteins identified and appeared to label a broader range of PM-associated proteins compared to the glycocapture strategy. While aminooxy-biotin effectively targets sialylated glycoproteins, resulting in very specific labelling of cell surface proteins, the higher yield from combinatory labelling of both amine-reactive and carboxyl-reactive functional groups using SUCAM is probably due to the higher abundance of primary amines (lysine residues and N-termini) and carboxyl residues available for labelling, compared to the specific glycosylation sites. Therefore, while aminooxy-biotin provides a very clean, targeted subset of the membrane proteome, SUCAM provides a more comprehensive set of surface proteins identified with higher counts.

In addition, SUCAM identified and quantified novel surface targets using Absolute units (copy numbers). Absolute determination of surface copy number is a new class-specific target requirement to channel targets to prosecution by different immunotherapy modalities, where high copy number targets (>20K to 100K per cell) are suitable for ADC approaches whereas lower surface copy numbers, but with retained differential over normal tissue surface abundance, are more suited to cellular effector cytotoxicity, either through direct recombination of cytotoxic cells in CAR-Ts or recruitment of native CTLs via bispecific antibodies (*e*.*g*.*,* AMG757 - successfully re-targeting DLL3 with a DLL3-CD3 BiTe) (34).

In conclusion, in this study we developed and investigated the efficacy of a multiplexed biotin derivatization strategy for cell surface proteome enrichment, which we named SUCAM. The approach consists of labelling with two amine reactive agents simultaneously followed by carboxyl labelling in one reaction. SUCAM increased the number of cell surface associated proteins identified and these were quantified in Absolute units (copy numbers) with good reproducibility. As SUCAM can uncover a wider scope of PMPs and enable absolute determination of surface copy number from low sample amounts, we expect this protocol will have broad applications in surfaceomic studies, including the discovery of novel diagnostic markers and potential therapeutic targets for immunotherapy.

## Data Availability

The mass spectrometry proteomics data have been deposited to the ProteomeXchange Consortium via the PRIDE (35) partner repository with the dataset identifier PXD068779 and 10.6019/PXD068779.

## Reviewer Access Details

Log in to the PRIDE website using the following details:

Project accession: PXD068779

Token: 28Z5jNEFSt2x

Username: reviewer_pxd068779@ebi.ac.uk

Password: YAoiUFA9rat0

## Supplemental Data

This article contains supplemental data.

## Conflictof Interests

P. R. C. and A. L. are the inventors of the SUCAM technology (patent pending). The other authors declare no competing interests.
