# Supplementary material for: Surfaceome Capture by Multiplex Biotinylation Enables Enhanced Identification of Cell Surface Proteins by Mass Spectrometry
Source: Mol Cell Proteomics. 2026 Apr 22;25(6):101572. doi: 10.1016/j.mcpro.2026.101572 (PMC13202573; doi:10.1016/j.mcpro.2026.101572)
Supplement: Supplemental Materials [file mmc14.docx]

**Supplementary Materials**

**Surfaceome Capture by Multiplex biotinylation (SUCAM) enables enhanced identification of cell surface proteins by mass spectrometry**

Ana Levi, Tommy Shields, Irbaz I.Badshah, Vinothini Rajeeve and Pedro R. Cutillas

Table of Contents

1. Supplementary Figures & Legends

Figure S1. Absolute numbers and overlaps of the cell surface associated proteome obtained by SUCAM and other biotin conjugation strategies

Figure S2. Pearson correlation data analysis

Figure S3. Comparing biotinylated surfaceome with total lysate proteome and total biotinylated proteome in SUCAM

Figure S4. Evaluation of cell surface- associated protein abundances across five biotin conjugation method

Figure S5. Quantitative Summary of the GO terms enriched across GO categories; BP, MF, CC in SUCAM

Figure S6. Western blot of cell surface proteins isolated from p31/Fuji using SUCAM

1. **Supplementary Table Legends**

Table S1: Sample and Data Relationship file (SDRF-Proteomics)

Table S2: Proteomics Dynamic Range Standard Set (UP2 protein standards) for Absolute Quantification

Table S3: Peptide and protein Identifications for cell surface proteins associated with Amine reactive Biotinylation, related to Fig. 3

Table S4: Peptide and protein identifications for cell surface proteins associated with Amine Reactive vs Carboxyl Reactive biotinylation, related to Fig.4

Table S5: Cell surface proteomic data associated with method comparison, related to Fig. 5 and Fig.6

Table S6: Protein and peptide identifications for cell surface proteomic data associated with method comparisons, related to Fig. 5

Table S7: Cell surface proteomic data associated with method comparisons, related to FigS1

Table S8: Protein Quantification data (Normalised. Intensities) for cell surface proteomic data associated with method comparison, related to Figure 7

Table S9: Protein Quantifications (Absolute Quantifications) for cell Surface proteins associated with SUCAM for AML cell lines, related to Fig.8

Table S10: Peptide and protein identifications for cell surface proteomics data associated with SUCAM for 7 AML cell lines, related to Fig. 8

Table S11: Quantitative Summary of GO terms enriched across GO categories; BP, MF, CC in SUCAM, related to S5

Table S12: Surfaceomic data associated with SUCAM and Glycoprotein capture comparison, related to Fig.10

Table S13: Peptide and Protein identifications for Surfaceomic proteins associated with SUACM vs Glycoprotein biotinylation, related to Fig.9&10

1. Supplementary Resources Table


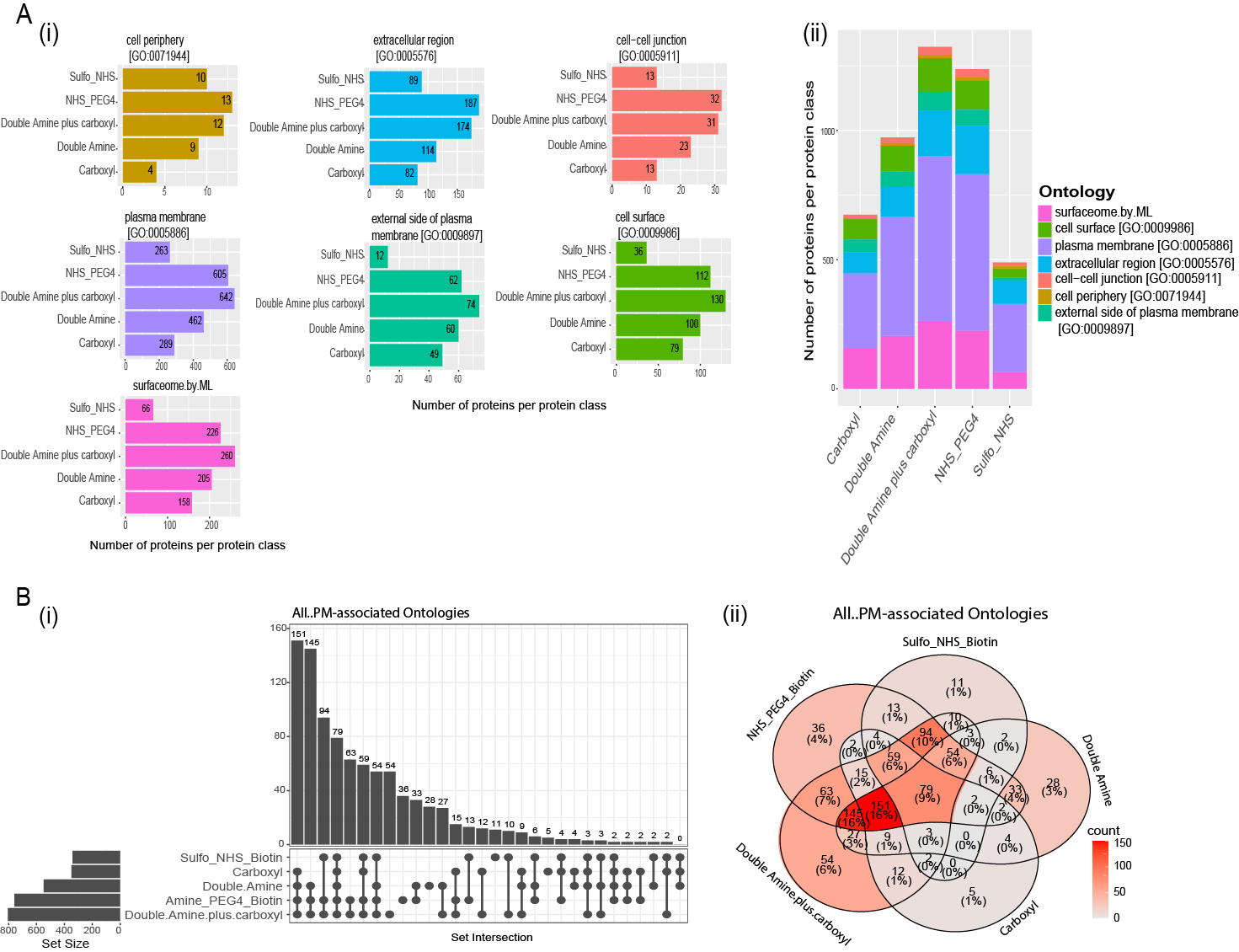


**Figure S1|** **Absolute numbers and overlaps of the cell surface associated proteome obtained by SUCAM and other biotin conjugation strategies.**

**A.** The proposed SUCAM (a.k.a. double amine plus carboxyl) approach was compared to other methods for the isolation of proteins annotated to be present in a wider cohort of cell surface associated ontologies. SUCAM (a.k.a. double amine plus carboxyl) was superior to other methods for detection of quantifiable proteins (Log_2_FC ≥ 0, p <0.1 were considered statistically significant) annotated to be present in a wider cohort of cell surface-associated ontologies. Ontologies were obtained from Uniprot and from Bausch-Fluck et al (27).

**B. (i)** An Upset plot and **(ii)** Venn Diagram showing percentages and overlaps of proteins annotated across ALL.plasma membrane associated GO terms show the highest number of absolute proteins (n= 811) to be captured by Double amine plus carboxyl and 6% of this protein repertoire was unique to SUCAM. N = 2 independent replicates, and X2 MS technical replicates (injections) were conducted for each method.


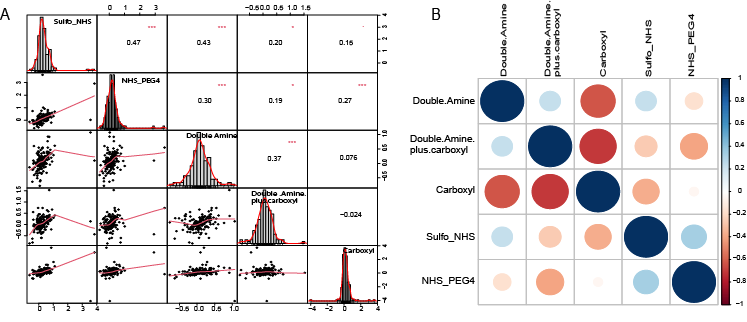
**Figure S2|** **Correlation data analysis.** Pearson's coefficient quantifies the strength and direction of the correlation between the five biotin cross-linking conditions in 1.2 mullion p31/Fuji cells.

**A.** A chart representing the correlation matrix provides scatter plots (lower triangles) and correlation values (upper triangles). Negative correlation, as shown by a more negative correlation coefficient value (-0.024) was observed between double amine plus carboxyl and Carboxyl, whilst a moderately positive correlation coefficient (0.37) was observed between Double amine plus carboxyl and double amine. A relatively weak correlation (0.19, 0.20) was observed between double amine plus carboxyl and NHS-PEG4 and Sulfo-NHS, respectively. Significance levels (p-values) were calculated using cor.test() and were used to determine if the correlations were statistically significant. Significance level thresholds; (*p* < 0.05 (*), *p* < 0.01 (**), *p* < 0.0001 (****)

**B.** The Pearson’s correlation matrix is represented as a Correlogram depicting inter-relationship between five different biotin conjugation methods used for this study. Positive correlations are displayed in blue and negative correlations in red colour. Colour intensity and the size of the circle are proportional to the correlation coefficients. In the right side of the correlogram, the legend colour shows the correlation coefficients and the corresponding colours. Corrplot R package was used for Pearson’s correlation coefficient analysis and visualisation. The function chart. Correlation() in the package Performance Analytics was used to display the chart of a correlation matrix. N = 2 independent replicates, and X2 MS technical replicates (injections) were conducted for each method.


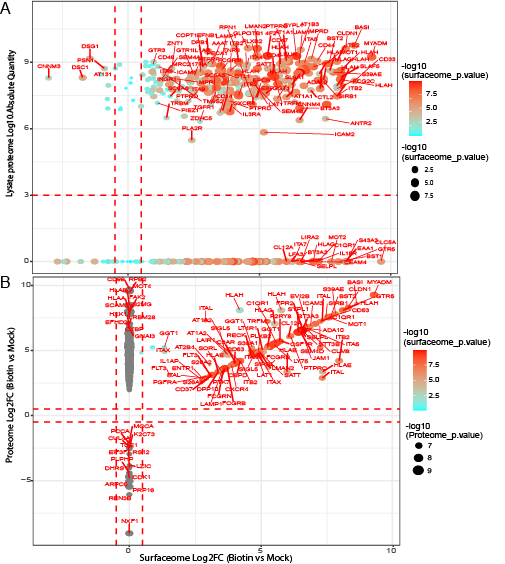


**Figure S3|** **Comparing biotinylated surfaceome with total lysate proteome and total biotinylated proteome in SUCAM**.

**A.** A scatterplot representing the relationship between the Log₂FC (Biotin vs mock) of the Surfaceome captured by SUCAM and the Log10.absolute quantification of the total proteome from total cell lysates shows an overlap of 20% (Log₂FC > |0.5| for surfaceome data, log10-absolute quantification for lysate proteomic data > 3).

**B.** Comparison of surfaceome and proteome log₂ fold changes for overlapping proteins shows 47% of surfaceome to be primarily detected in the proteomic data (log₂-fold change > |0.5| for proteome and surfaceome data). N = 2 independent replicates, and X2 MS technical replicates (injections) were conducted for each method.


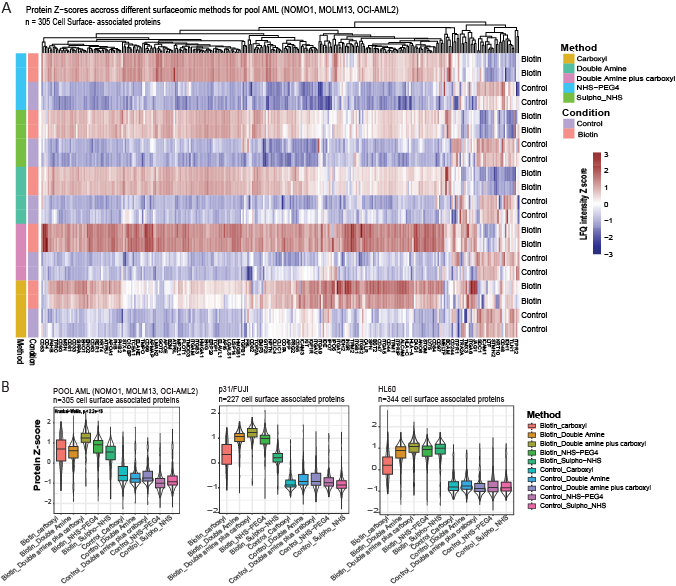


**Figure S4|** **Evaluation of cell surface- associated protein abundances across five biotin conjugation methods.**

**A** Heatmap showing expression levels (z scores of median Log2.Normalised.Intensities) of cell surface proteins depicts changes in the abundance of labelled cell surface proteins across five conditions identified relative to mock unlabelled control. N = 3 independent replicates, and X2 MS technical replicates (injections) were conducted for each method for pool of 3 AML cell lines (NOMO1, MOLM13, OCI-AML2).

**B.** Normalised intensities of cell surface-associated proteins across the named conditions. Kruskal-Wallis test shows a significantly (p < 2.2 x 10^-16^) higher expression of cell surface associated proteins in the double amine plus carboxyl compared to the other labelling methods. N = 3 independent replicates, and X2 MS technical replicates (injections) were conducted for each method for POOL of 3 AML cell lines (NOMO1, MOLM13, OCI-AML2), p31/Fuji and HL60.


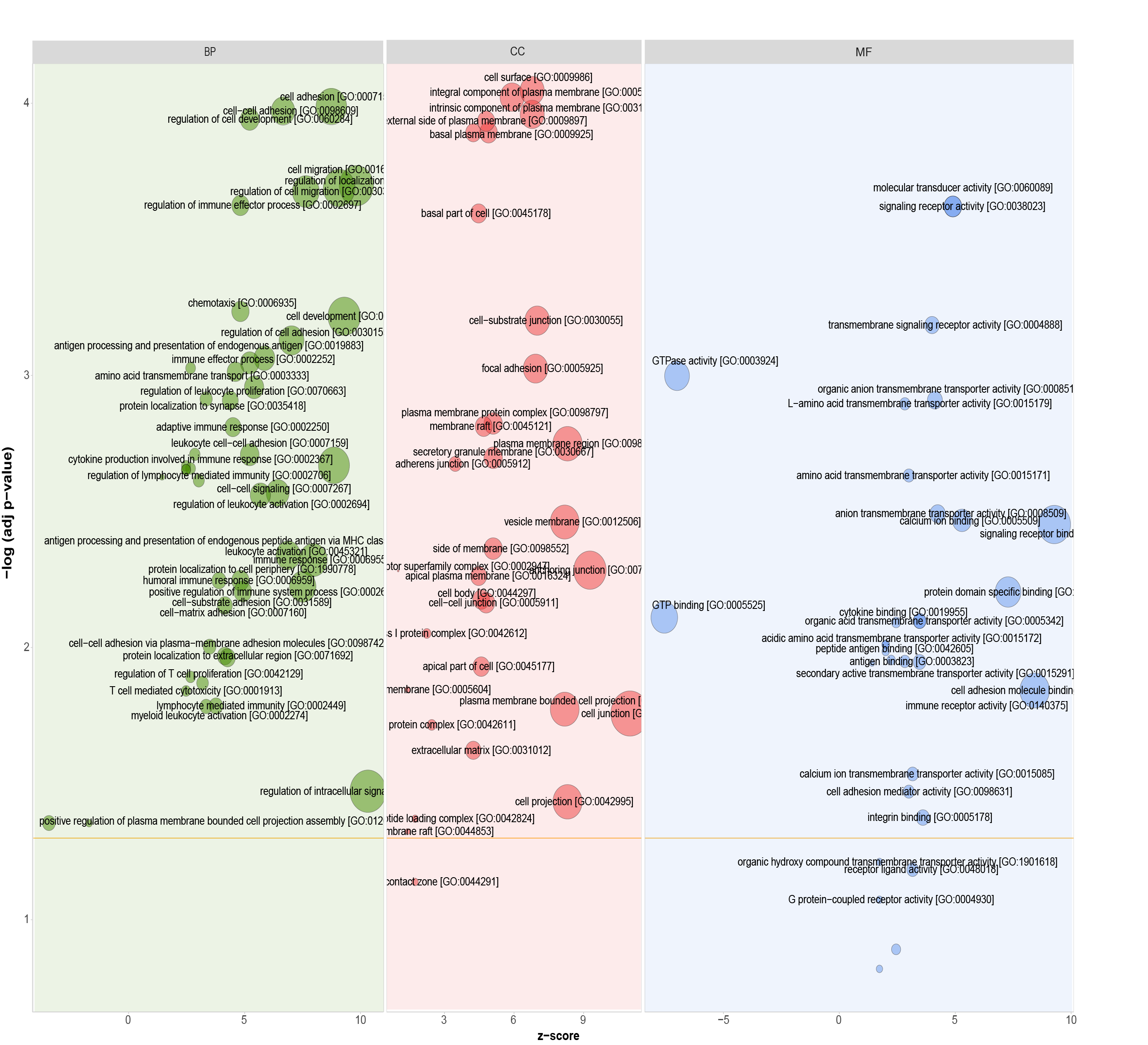


**Figure S5|** **Quantitative Summary of the GO terms enriched across GO categories; BP, MF, CC in SUCAM.**

GO enrichment across BP, CC and MF categories is quantitively and visually summarised as a GOBubble plot. The enrichment of GO terms across BP, CC and MF categories for the proteins differentially expressed in biotin compared to mock for SUCAM. GSEA was used to perform a gene- annotation enrichment analysis of the set of differentially expressed (Biotin vs Control) genes (Log₂FC ≥ 0, Benjamini-Hochberg adjusted p-value < 0.1). The data set contains the five following items: The x-axis represents the z-score, and the y-axis represents the -log10(p-value) of different GO items. The size of the bubble indicates the number of genes enriched for each GO term. The different coloured bubble plots represent different GO categories. N = 6 independent replicates were conducted for each method, and each sample was injected twice (x2) into the system for MS.


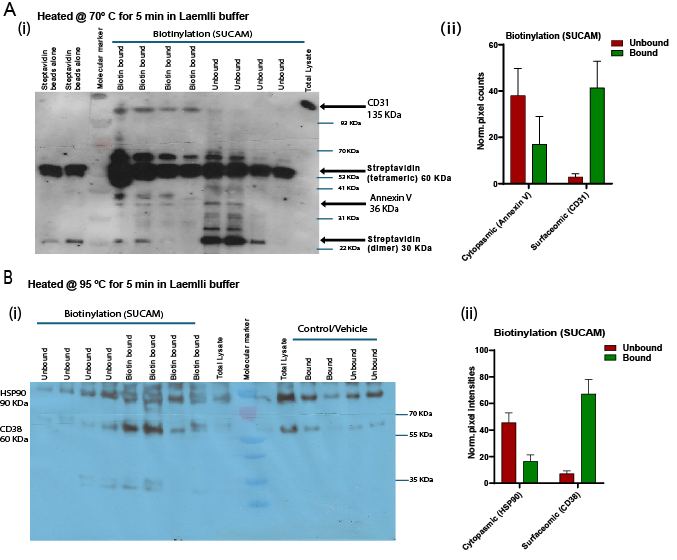


**Figure S6|** **Western blot of cell surface proteins isolated from p31/Fuji using SUCAM. A. (i)** AML cell line, p31/Fuji were biotin labelled using SUCAM methodology and the biotinylated proteins were isolated using Streptavidin Agarose Resin. Streptavidin resin alone (positive control), proteins eluted from the Streptavidin resin (biotin bound proteins) and unbound proteins (non-biotinylated) were analysed by western blotting from samples prepared using mild denaturing conditions (i.e. heating at 70°C). Under these conditions, streptavidin is exceptionally stable and remains as an intact, tetrameric form (approx. 52–60 kDa) rather than fully dissociating into its individual monomers (approx. 13–15 kDa). Unbound fraction represents proteins that did not bind to the Streptavidin matrix during incubation. Western blot was cut at the 70 KDa mark. The top membrane (>70KDa) was probed with ant-CD31 Antibody, detecting CD31(cell surface protein) at approximately 135 KDa while the bottom membrane (<70 KDa) was probed with anti-Annexin V Antibody, detecting Annexin V (intracellular protein) at approximately 36 KDa. The cell surface protein CD31 is detected in the bound fraction of the sample treated with biotinylating reagents but not in the unbound fraction. In contrast, Annexin is mostly observed in the unbound fraction with less expression in the bound fraction. **(ii)** Relative quantification of protein bands from western blotting using ImageJ.

**B. (i)** AML cell line, p31/Fuji were biotin labelled using SUCAM methodology or using vehicle/control. The SUCAM labelled biotinylated proteins and vehicle (unlabelled) proteins were isolated using Streptavidin Agarose Resin. Samples were prepared for western blotting using strongly denaturing conditions (i.e. heating at 95 °C) so that Streptavidin (50-60 kDa) dissociates into ~15 kDa monomers when heated or in harsh SDS conditions (15 kDa monomers run off the gel, hence not evident). Total cell lysate, proteins eluted from the Streptavidin beads from both SUCAM biotin labelled and vehicle unlabelled fraction and their unbound counterparts (proteins that did not bind to the Streptavidin matrix) were analysed by western blotting for human CD38 (cell surface protein) and HSP90 (intracellular protein). Western blot was cut at the 70 KDa mark. The top membrane (>70KDa) was probed with ant-HSP90 Antibody, detecting HSP90 at approximately 90 KDa while the bottom membrane (<70 KDa) was probed with anti-CD38 Antibody, detecting CD38 (glycosylated) at approximately 60 KDa. The cell surface protein CD38 is detected in the bound fraction of the sample treated with biotinylating reagents but not in the sample treated with vehicle/control. In contrast, HSP90 is observed in the unbound fraction of both biotinylated and control samples. **(iii)** Relative quantification of protein bands from western blots using ImageJ.

| **REAGENT or RESOURCE** | **SOURCE** | **IDENTIFIER** |
| --- | --- | --- |
| **BIOTINYLATING REAGENTS** | | |
| EZ-Link Sulfo-NHS-SS-Biotin | ThermoFisher Scientic (Pierce) | 21331 |
| EZ-Link NHS-PEG4-Biotin | ThermoFisher Scientic (Pierce) | 21330 |
| EX-Link-Amine PEG2 Biotin | ThermoFisher | 21346 |
| NHS-Sulfo | ThermoFisher | 82072 |
| EDC | ThermoFisher Scientic (Pierce) | 22980 |
| Pierce Streptavidin Magnetic Beads | Thermo Fisher Scientific | 88817 |
| Pierce™ High Capacity Streptavidin Agarose | Thermo Fisher Scientific | 20357 |
| Alkoxyamine-PEG4-biotin | Thermo Fisher Scientific | 26137 |
| Aniline | Sigma-Aldrich | 242284 |
| GlycoLink™ Immobilization Kit | Thermo Fisher Scientific | 88941 |
| Proteomics Dynamic Range Standard Set | Sigma-Aldrich | UPS2 |
| **CHEMICALS** | | |
| RIPA Lysis and Extraction Buffer | ThermoFisher Scientic (Pierce) | 89900 |
| EDTA | Sigma Aldrich | E7889 |
| Trypsin/Lys-C Protease Mix | ThermoFisher Scientific | A40007 |
| RPMI 1640 Medium | ThermoFisher Scientific | 61870010 |
| DMEM, high glucose | ThermoFisher Scientific | 31966021 |
| MEM alpha | ThermoFisher Scientific | 22571020 |
| Opti-MEM Reduced Serum Medium | ThermoFisher Scientific | 31985062 |
| Heat Inactivated Fetal Bovine Serum | ThermoFisher Scientific | 10500-064 |
| Penicillin-Streptomycin (10,000 U/mL) | ThermoFisher Scientific | 15140122 |
| Phosphate Buffered Saline (PBS) | Sigma-Aldrich | D8537 |
| Sodium (meta)periodate | Sigma-Aldrich | S1878 |
| Sodium Fluoride (NaF) | Sigma-Aldrich | 201154 |
| Sodium Orthovanadate (Na_3_VO_4_) | Sigma-Aldrich | 450243 |
| Sodium β-glycerophosphate | Sigma-Aldrich | G9422 |
| Sodium pyrophosphate (Na_4_P_2_O_7_) | Sigma-Aldrich | 71501 |
| Urea | Sigma-Aldrich | U5378 |
| HEPES sodium salt | Sigma-Aldrich | H7006 |
| DTT | ThermoFisher Scientific | R0862 |
| Iodoacetamide | Sigma-Aldrich | I1149 |
| Water for LC-MS (Optigrade) | LGC | SO-9368-B025 |
| Acetonitrile for LC-MS (Optigrade) | LGC | SO-9340-B025 |
| Trifluoroacetic acid UHPLC-MS (Optigrade) | LGC | SO-9668-B001 |
| Glycolic acid | Fisher Scientific | 10746561 |
| Ammonium acetate | Sigma-Aldrich | 09689 |
| Ammonia solution | LGC Standards | HPA-0070-B010 |
| Formic acid | ThermoFisher Scientific | F-1850-PB08 |
| Enolase | Waters | 186002325 |
| Guava ViaCount reagent | Merck Millipore | 4000-0040 |
| Protease inhibitor cocktail | Sigma-Aldrich | P8340 |
| PMSF | Sigma-Aldrich | 93482 |
| Okadaic acid | Sigma-Aldrich | O7885 |
| Glycine | ThermoFisher Scientific | A18822.14 |
| Ammonium bicarbonate | Sigma Aldrich | A6141 |
| **WESTERN BLOTTING REAGENTS** | | |
| NuPAGE 4-12% Bis-Tris Protein Gels, 1.0 mm | ThermoFisher Scientific | NP0326BOX |
| NuPAGE MOPS SDS running buffer 20x | ThermoFisher Scientific | NP0001 |
| NuPAGE LDS sample buffer 4x | ThermoFisher Scientific | NP0007 |
| NuPAGE Transfer buffer 20x | ThermoFisher Scientific | NP0006 |
| NuPAGE Antioxidant | ThermoFisher Scientific | NP0005 |
| iBlot Transfer Stack, nitrocellulose | ThermoFisher Scientific | IB301001 |
| SuperSignal West Pico Plus | ThermoFisher Scientific | 34577 |
| Pierce BCA protein assay kit | ThermoFisher Scientific | 23227 |
| Biotin | Sigma Aldrich | B4501 |
| IGEPAL-CA630 | Sigma Aldrich | I8896 |
| [CD31 (PECAM-1) (F8M3S) Rabbit Monoclonal Antibody](https://www.cellsignal.com/products/primary-antibodies/cd31-pecam-1-f8m3s-rabbit-monoclonal-antibody/95462) | Cell Signaling Technologies | #95462 |
| Annexin V (E3W8V) Rabbit Monoclonal Antibody | Cell Signaling Technologies | #54043 |
| CD38 (E7Z8C) Rabbit Monoclonal Antibody | Cell Signaling Technologies | #51000 |
| HSP90 Rabbit Antibody | Cell Signaling Technologies | #4874 |
| Anti-rabbit IgG, HRP-linked Antibody | Cell Signaling Technologies | #7074 |
| **Mass Spec Prep Material** | | |
| Oasis HLB 1cc cartridge, 10 mg | Waters | WAT094225 |
| Titansphere beads | GL Sciences | 5020-75010 |
| PE-filtered spin tips | Glygen | TF2EMT |
| Immobilized Trypsin, TPCK treated | ThermoFisher Scientific | 20230 |
| Eppendorf Protein LoBind tubes, 1.5 mL | Sigma-Aldrich | Z666505-100EA |
| Eppendorf Protein LoBind tubes, 2 mL | Sigma-Aldrich | Z666513-100EA |
| EASY-Spray source | ThermoFisher Scientific | ES081 |
| µ-pre-column Acclaim PepMap 100 C18 LC | ThermoFisher Scientific | 160454 |
| Analytical column Acclaim PepMap 100 C18 LC | ThermoFisher Scientific | 164569 |
| **EXPERIMENTAL MODELS: CELL LINES** | | |
| Human: HL60 | ATCC | CCL-240 |
| Human: KASUMI-1 | ATCC | CRL-2724 |
| Human: P31/FUJ | JCRB | 0091 |
| Human: THP-1 | ATCC | TIB-202 |
| Human: MOLM13 | DSMZ | ACC-554 |
| Human: NOMO-1 | DSMZ | ACC-542 |
| Human: OCI-AML2 | DSMZ | ACC-99 |
| Human:JHH4 | ATCC | CVCL_2787 |
| **CELL CULTURE MATERIALS** | | |
| RPMI 1640 Medium | ThermoFisher Scientific | 61870010 |
| DMEM, high glucose | ThermoFisher Scientific | 31966021 |
| MEM alpha | ThermoFisher Scientific | 22571020 |
| Opti-MEM Reduced Serum Medium | ThermoFisher Scientific | 31985062 |
| Heat Inactivated Fetal Bovine Serum | ThermoFisher Scientific | 10500-064 |
| 10X Collagenase/Hyaluronidase | Stem Cell Technologies | #07912 |
| DNAse I solution | Stem Cell Technologies | #07900 |
| Dulbecco’s Modified Eagle’s Medium (DMEM), low glucose | Sigma Aldrich | [D5523](https://www.sigmaaldrich.com/GB/en/product/sigma/d5523) |
| Ammonium Chloride Solution | Stem Cell Technologies | #07800 |
| **SOFTWARE AND ALGORITHMS** | | |
| Xcalibur | ThermoFisher Scientific | https://www.thermofisher.com/search/results?query=xcalibur%E2%84%A2&navId=12141&persona=Catalog |
| Mascot Daemon (2.5.0) | Matrix Science | http://www.matrixscience.com/ |
| Mascot Distiller (v2.5.1.0) | Matrix Science | http://www.matrixscience.com/ |
| Mascot Search engine (v2.5) | Matrix Science | http://www.matrixscience.com/ |
| Pescal | In-house | https://www.mcponline.org/content/6/9/1560.long |
| Gephi (v0.9.1) | Gephi | <https://gephi.org/> |
| CytoSoft (v2.5.7) | Luminex | <https://www.luminexcorp.com/guava-easycyte-software/> |
| Image Studio Lite (v5.2) | LI-COR | <https://www.licor.com/bio/image-studio-lite/download> |
| RStudio Desktop (v1.2.5033) | RStudio | https://rstudio.com/ |
| ggplot2 package (v3.3.1) | CRAN r project | <https://cran.r-project.org/web/packages/ggplot2/index.html> |
| Prism 8 | GraphPad | <https://www.graphpad.com/scientific-software/prism/> |
| Adobe Illustrator | Adobe | <https://www.adobe.com/uk/products/illustrator.html> |
| **Deposited data** | | |
| Mass spectrometry Proteomic Data | This study | PRIDE: PXD068779 |
